# Single-Strand Annealing Plays a Major Role in Double-Strand DNA Break Repair following CRISPR-Cas9 Cleavage in *Leishmania*

**DOI:** 10.1128/mSphere.00408-19

**Published:** 2019-08-21

**Authors:** Wen-Wei Zhang, Greg Matlashewski

**Affiliations:** a Department of Microbiology and Immunology, McGill University, Montreal, Quebec, Canada; Stanford University

**Keywords:** CRISPR-Cas9, DNA polymerase theta, *Leishmania*, SaCas9, double-strand DNA break repair, gene deletion, gene targeting, microhomology-mediated end joining, miltefosine transporter gene, nonhomologous end joining, parasite, single-strand annealing

## Abstract

CRISPR-Cas9 genome editing relies on an efficient double-strand DNA break (DSB) and repair. Contrary to mammalian cells, the protozoan parasite *Leishmania* lacks the most efficient nonhomologous end-joining pathway and uses microhomology-mediated end joining (MMEJ) and, occasionally, homology-directed repair to repair DSBs. Here, we reveal that *Leishmania* predominantly uses single-strand annealing (SSA) (>90%) instead of MMEJ (<10%) for DSB repair (DSBR) following CRISPR targeting of the miltefosine transporter gene, resulting in 9-, 18-, 20-, and 29-kb sequence deletions and multiple gene codeletions. Strikingly, when targeting the Leishmania donovani LdBPK_241510 gene, SSA even occurred by using direct repeats 77 kb apart, resulting in the codeletion of 15 *Leishmania* genes, though with a reduced frequency. These data strongly indicate that DSBR is not efficient in *Leishmania*, which explains why more than half of DSBs led to cell death and why the CRISPR gene-targeting efficiency is low compared with that in other organisms. Since direct repeat sequences are widely distributed in the *Leishmania* genome, we predict that many DSBs created by CRISPR are repaired by SSA. It is also revealed that DNA polymerase theta is involved in both MMEJ and SSA in *Leishmania*. Collectively, this study establishes that DSBR mechanisms and their competence in an organism play an important role in determining the outcome and efficacy of CRISPR gene targeting. These observations emphasize the use of donor DNA templates to improve gene editing specificity and efficiency in *Leishmania*. In addition, we developed a novel Staphylococcus aureus Cas9 constitutive expression vector (pLdSaCN) for gene targeting in *Leishmania*.

**IMPORTANCE** Due to differences in double-strand DNA break (DSB) repair mechanisms, CRISPR-Cas9 gene editing efficiency can vary greatly in different organisms. In contrast to mammalian cells, the protozoan parasite *Leishmania* uses microhomology-mediated end joining (MMEJ) and, occasionally, homology-directed repair (HDR) to repair DSBs but lacks the nonhomologous end-joining pathway. Here, we show that *Leishmania* predominantly uses single-strand annealing (SSA) instead of MMEJ for DSB repairs (DSBR), resulting in large deletions that can include multiple genes. This strongly indicates that the overall DSBR in *Leishmania* is inefficient and therefore can influence the outcome of CRISPR-Cas9 gene editing, highlighting the importance of using a donor DNA to improve gene editing fidelity and efficiency in *Leishmania*.

## Abstract (precis)

Due to differences in double-strand DNA break (DSB) repair mechanisms, CRISPR-Cas9 gene editing efficiency can vary greatly in different organisms. In contrast to mammalian cells, the protozoan parasite *Leishmania* uses microhomology-mediated end joining (MMEJ) and, occasionally, homology-directed repair (HDR) to repair DSBs but lacks the nonhomologous end-joining pathway. Here, we show that *Leishmania* predominantly uses single-strand annealing (SSA) instead of MMEJ for DSB repairs (DSBR), resulting in large deletions that can include multiple genes. This strongly indicates that the overall DSBR in *Leishmania* is inefficient and therefore can influence the outcome of CRISPR-Cas9 gene editing, highlighting the importance of using a donor DNA to improve gene editing fidelity and efficiency in *Leishmania*.

## INTRODUCTION

*Leishmania* protozoans cause a spectrum of diseases ranging from mild cutaneous infection to severe visceral leishmaniasis, which can be fatal if it is not treated. Despite decades of research, there is still no effective vaccine, and the treatment of leishmaniasis relies on chemotherapy with significant side effects (1). *Leishmania*, an early-branching lower eukaryote, contains 34 to 36 chromosomes with a genome size of 33 Mb encoding about 8,000 genes; more than half of these genes encode hypothetical proteins with no known function. Although *Leishmania* is considered an asexual and diploid organism, aneuploidy is frequently observed for many of its chromosomes (2–9). Most *Leishmania* genes contain no intron and are separated with variable-length intergenic sequences. *Leishmania* transcribes its genes as long polycistronic units, and expression of each individual gene is mainly controlled by copy number variation and posttranscription and translation control (2–6). In particular, the direct or inverted repeat sequences, which are widely distributed in the intergenic sequences in the *Leishmania* genome and which are conserved in *Leishmania* species, are often used to form extrachromosomal circular or linear amplicons for gene amplification (or deletion) to meet its changing environment, such as exposure to drugs (10, 11). An RNA interference (RNAi) pathway is not present in most *Leishmania* species (12).

Gene targeting in *Leishmania* used to rely on homologous recombination (HR) with selection markers (13–16). CRISPR-Cas9 has significantly improved gene editing efficiency in *Leishmania* (17–23). However, compared with the editing frequency (30% to 90%) in mammalian cells and other organisms, such as Toxoplasma gondii (24–26), the CRISPR gene-targeting efficiency is low in *Leishmania*, with a mutation frequency of less than 1% within 2 weeks after CRISPR reagent transfection (18, 19). Consequently, an antibiotic selection marker donor is typically required to improve the identification of CRISPR gene deletion or disruption mutants in *Leishmania* (17–23).

CRISPR-Cas-mediated genome editing relies not only on the generation of specific DNA double-strand breaks (DSB) by the Cas nuclease but also on the efficient repair of these DSBs through the cellular machinery (27). DSBs are lethal to the cell and must be repaired to restore the integrity and functionality of the genome. In mammalian cells, DSBs are repaired through one of the DSB repair (DSBR) pathways, namely (in the order of usage frequency), nonhomologous end joining (NHEJ), homology-directed repair (HDR), microhomology-mediated end joining (MMEJ), and single-strand annealing (SSA) (27–30). Each of these pathways requires specific repair factors, is active at different rates at different phases of the cell cycle, and often produces very different repair outcomes. NHEJ requires the presence of the heterodimeric DNA-binding complex Ku70/80 and DNA ligase IV and involves direct ligation at the break site, often with small insertions or deletions. NHEJ typically acts first to attempt to repair DSBs (31–32) and is much faster than HDR, taking place within 30 min (versus 7 h or longer for HDR), and accounts for ∼75% of repair events (32). If NHEJ cannot be completed, then the DSB undergoes 5′-to-3′ resection to produce 3′ single-stranded DNA (ssDNA) overhangs suitable for alternative pathways of repair (33, 34). If the cell cycle is in S phase, HDR involving RAD51 uses the homology sequence present in the sister chromatid as a repair template for the DSB. HDR is usually considered a faithful pathway, though it may occasionally contribute to mutation. Depending on the cell type, HDR accounts for 25 to 30% of total repair events (31). MMEJ and SSA are seldom used for DSBR and are considered the backup pathways when the NHEJ and HDR pathways are absent or disrupted, such as in some cancer cells (33–37). MMEJ, as the name indicates, uses the microhomology sequences (5 to 25 nucleotides [nt]) present in each of the 3′ overhangs to anneal ssDNA sequences. Processing and excision of the intervening sequence result in deletions of various sizes at the repair junction, depending on the distance between the microhomology sequences. DNA polymerase theta (Polθ) plays a central role in MMEJ, and therefore, MMEJ is also called DNA polymerase theta-mediated end joining (TMEJ) (36–41). SSA requires the presence of long homologous direct repeat sequences (26 to 500 bp) flanking the DSB and extensive resections. Annealing can take place between the complementary sequences located on opposite sides of the DSB. This is followed by the nucleolytic removal of any remaining tails, DNA synthesis to fill in gaps, and ligation. SSA often results in large deletion mutations and causes the most severe damage to the genome. While SSA shares some repair factors with MMEJ, SSA also requires specific factors, such as RAD52, to complete the DSBR (42–46).

Contrary to mammalian cells and other organisms, the most efficient NHEJ pathway is not present in *Leishmania* because it lacks the key NHEJ repair cofactors DNA ligase IV and XRCC4 (2, 47–50). Although *Leishmania* is a diploid organism and gene targeting once depended on homologous recombination, HDR is seldom used for DSBR (19). MMEJ plays an important role in DSBR in *Leishmania* (18, 19). In this study, by examining individual clones of CRISPR-targeted *Leishmania* cells, we found that with the use of the direct repeat sequences flanking the Cas9 cleavage sites, SSA instead of MMEJ is the main mechanism of DSBR for CRISPR targeting of the miltefosine transporter (*MT*) gene. Because of DSBR by SSA, 9- to 77-kb genomic sequence deletions were observed, strongly indicating that MMEJ and the overall DSBR are not efficient in *Leishmania*. This helps explain why CRISPR gene-targeting efficiency is low and why no single guide RNA (gRNA) design programs tested in this study could correctly predict gRNA activity in *Leishmania*. We developed a novel constitutive Staphylococcus aureus Cas9 (SaCas9) expression vector which has a gene-targeting efficiency similar to that of the Streptococcus pyogenes Cas9 (SpCas9) vector. In addition, we demonstrate that DNA polymerase theta plays an important role in MMEJ as well as SSA and provide evidence that CRISPR-Cas9-generated DSBs may promote a linear chromosome duplication in *Leishmania*.

## RESULTS

### No single current gRNA design program tested can accurately predict gRNA activity in *Leishmania*, and the gene-targeting efficiency for the same gRNA construct can vary greatly in different *Leishmania* species.

**FIG 1:**
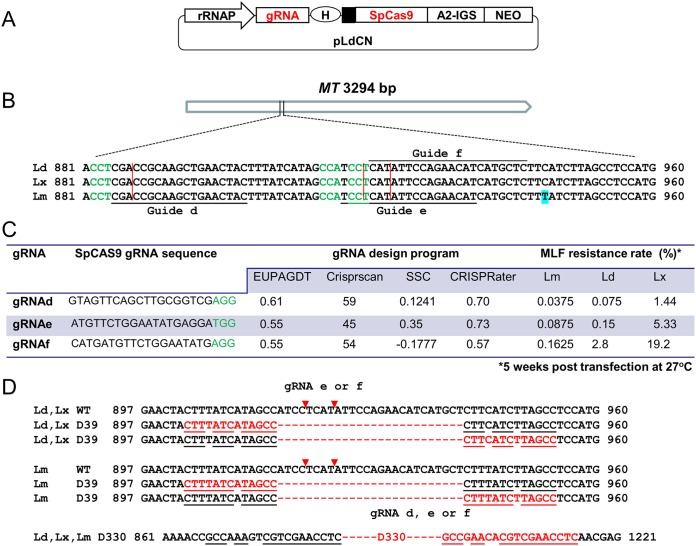
Gene-targeting efficiency of 3 SpCas9 gRNA constructs in L. donovani (Ld), L. major (Lm), and L. mexicana (Lx). (A) Schematic of the *Leishmania* CRISPR vector pLdCN, which coexpresses SpCas9 and its gRNA under the control of the L. donovani rRNA promoter (rRNAP) (19). H, hepatitis delta virus (HDV) ribozyme. The small black box upstream of Cas9 represents the 92-bp pyrimidine track. A2-IGS, *A2* intergenic sequence; NEO, neomycin resistance gene. The drawing is not to scale. (B) The conserved target sites of SpCas9 gRNAd, gRNAe, and gRNAf in the *MT* genes of three *Leishmania* species. Note that only the complementary sequences of these gRNA target sites are shown. (C) The gRNA activity scores predicted by 4 different gRNA design programs and the actual gene-targeting efficiencies, expressed as MLF resistance rates, in 3 different *Leishmania* species. Note that no single gRNA program could accurately predict the activity of these gRNAs in *Leishmania*. The triple nucleotides NGG, known as the protospacer-adjacent motif (PAM), are indicated in green for each SpCas9 gRNA. (D) Deletions resulting from MMEJ detected in the *Leishmania* species after gRNAd, -e, and -f targeting. The red letters and dashed line represent deleted sequences. The Cas9 cleavage sites are marked with either red vertical lines (B) or arrowheads (D).

Since most gRNA design programs (algorithms) are developed using data from mammalian cells, these may not be suitable for *Leishmania* (18). The SpCas9-expressing *Leishmania* CRISPR vector (pLdCN; Fig. 1A) previously developed uses the Leishmania donovani rRNA promoter to drive gRNA expression (19), and it is unknown whether this vector functions as well in other *Leishmania* species. We therefore attempted to identify a gRNA design program that can more accurately predict gRNA activity in *Leishmania* and determine whether the gene-targeting efficiency with the pLdCN vector is similar in different *Leishmania* species. Three gRNAs, termed gRNAd, gRNAe, and gRNAf, that target the identical sequences of the miltefosine transporter (MT) gene in L. donovani, L. major, and L. mexicana were designed (Fig. 1B). The *MT* gene was chosen as the target gene because the MT proteins on the cell membrane are required to transport extracellular miltefosine (MLF) into the cell, and a deletion, a disruption, and even a single point mutation in the *MT* gene can block MLF uptake, resulting in *Leishmania* resistance to MLF (18, 19, 51, 52). The *MT* gene has therefore been shown to be an ideal target gene model to study the efficiency and mechanism of CRISPR-mediated genome editing in L. donovani (18, 19). The relative activities of the above-described gRNAs that were designed (Fig. 1B) were assessed with four gRNA design programs, including EuPaGDT, CRISPRscan, Sequence Scan for CRISPR (SSC), and CRISPRater (53–56). These programs predict gRNA activity mainly based on the nucleotide composition of the gRNA guide sequence, though the off-target sites in a genome could also be included as the score parameter. A higher activity score would suggest that a gRNA could be more active in assisting Cas9 with generating the specific DSB. As shown in Fig. 1C, the three gRNAs received very different activity scores on varied scales from these gRNA design programs. Although a consensus activity order for these three gRNAs could be reached between SSC and CRISPRater, the scores obtained with the latter program were clearly more clustered than the ones obtained with SSC. The guide-coding sequences of these gRNAs were cloned into pLdCN vectors and transfected into L. donovani, L. major, and L. mexicana promastigotes. At 5 weeks posttransfection, the promastigotes were subjected to MLF selection, and survival rates were determined by limiting dilution culture in 96-well plates. The MLF resistance rate (the frequency of double *MT* gene allele mutation) provides an estimate of the activity of each gRNA in these *Leishmania* species (Fig. 1C). Similar to previous observations (18), the different gRNAs had different activities in the same *Leishmania* species. Although the target site for gRNAf was only a 4-bp shift from the gRNAe target site, there was a 2- to 18-fold difference in activity depending on the species. The gRNA activity order (gRNAf > gRNAe > gRNAd) was the same in the 3 *Leishmania* species, indicating that the gRNA sequence did have an influence on the gene editing efficiency. However, none of the four gRNA design programs was able to rank these gRNA activities correctly. For example, in contrast to the actual activity, EuPaGDT and CRISPRscan gave the highest activity score to gRNAd and SSC and CRISPRater ranked gRNAf the lowest. As also shown in Fig. 1C, the same gRNA could have very different activity in different *Leishmania* species, with the highest activity always being observed in L. mexicana and the lowest activity always being observed in L. major, with the difference being 40- to 120-fold. Together, these results show that no gRNA design program tested could correctly predict these gRNA activities and that the same gRNA construct could have different gene-targeting efficiencies in different *Leishmania* species, which could reflect the variation in gRNA and Cas9 expression and DSBR efficiency in these *Leishmania* species (see Discussion).

### Development of a constitutive Staphylococcus aureus Cas9 CRISPR vector for *Leishmania*.

**FIG 2:**
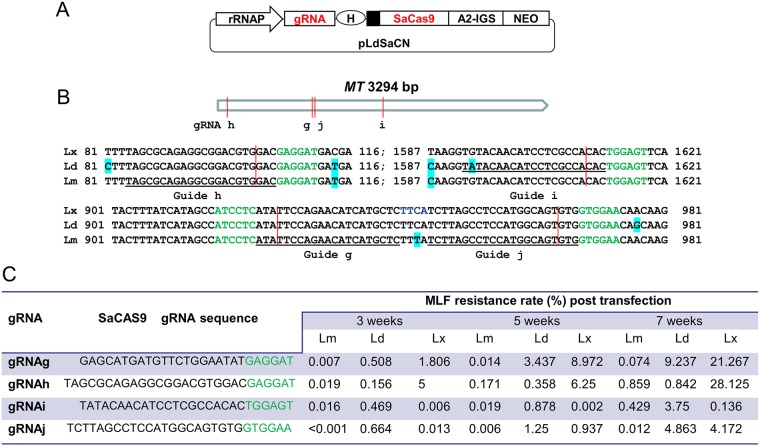
Development of the constitutive SaCas9 and gRNA coexpression vector pLdSaCN and its use for gene targeting in different *Leishmania* species. (A) Schematic of the *Leishmania* CRISPR vector pLdSaCN, which coexpresses SaCas9 and its gRNA under the control of the L. donovani rRNA promoter (rRNAP). Other abbreviations are defined in the Fig. 1A legend. The drawing is not to scale. (B) The conserved target sites of SaCas9 gRNAg, -h, -i, and -j in the *MT* genes of L. donovani (Ld), L. major (Lm), and L. mexicana (Lx). The letters in green represent the SaCas9 NNGRRT (N) PAM sequences; the SaCas9 cleavage sites are marked with red vertical lines. The nonconserved nucleotides are highlighted with bright green in the alignment of these 3 *Leishmania* sequences. Note that there was 1 nucleotide difference (the second nucleotide, A/G) between the guide sequence of gRNAi and its target site in L. mexicana and L. major. (C) The gene-targeting efficiencies of SaCas9 gRNAg, -h, -i, and -j, expressed as MLF resistance rates, in 3 different *Leishmania* species. L. donovani, L. major, and L. mexicana promastigotes were transfected with pLdSaCN CRISPR vectors expressing gRNAg, -h, -i, and -j and selected with G418. The MLF resistance rates were determined at 3, 5, and 7 weeks posttransfection.

Compared with the commonly used Streptococcus pyogenes Cas9 (SpCas9; 1,368 amino acids [aa]), the smaller Cas9 orthologue derived from Staphylococcus aureus (SaCas9), with 1,053 amino acids, has been reported to be more efficient in digesting target DNA *in vitro*, to have a similar gene editing activity in mammalian cells, but to have fewer off-target sites since it uses a longer protospacer-adjacent motif (PAM) sequence (NNGRRT) (25). The SaCas9 ribonucleoprotein has recently been shown to have a high genome editing efficiency in transiently transfected trypanosomatidic parasites, including *Leishmania* (21). To determine how SaCas9 would perform when constitutively expressed in *Leishmania*, we developed a novel SaCas9 CRISPR vector, pLdSaCN, by replacing SpCas9 from pLdCN (19) with SaCas9 and its corresponding gRNA coding sequences (Fig. 2A). To test this vector, four gRNAs (termed gRNAg, gRNAh, gRNAi, and gRNAj) targeting conserved sequences in the *MT* gene in L. donovani, L. major, and L. mexicana (Fig. 2B and C) were expressed from the pLdSaCN vector, and MLF resistance rates were measured at 3, 5, and 7 weeks posttransfection. As shown in Fig. 2C, we had the following interesting observations. First, like SpCas9 gRNA, the different SaCas9 gRNAs had quite different activities in the same *Leishmania* species, and the gene editing frequency accumulated with time, as observed in all three *Leishmania* species. Second, likely because the GTGGAA PAM sequence (instead of NNGRRT) for gRNAj was not optimal, gRNAj displayed the lowest activity among these four SaCas9 gRNAs in L. major. However, a relatively high editing efficiency for gRNAj could still be observed in L. donovani and L. mexicana, indicating that these two species are more tolerant of the NNGRRN PAM sequence, which would suggest that more active sites could be available for SaCas9 targeting in these species. Third, a single nucleotide mismatch between the gRNAi guide sequence and the DNA target site, the second nucleotide A/G difference in L. mexicana and L. major (Fig. 2B), dramatically inhibited gRNAi activity in L. mexicana, but no obvious adverse effect on gRNAi activity was observed in L. major. Fourth, like SpCas9 targeting, the gene editing efficiency for the same SaCas9 gRNA construct could be very different in these three *Leishmania* species, with the overall activity being in the order L. mexicana > L. donovani > L. major. For example, 30- to 300-fold differences for gRNAg and gRNAh were observed between L. mexicana and L. major. Finally, as described above, the gene editing efficiency could vary greatly among the different gRNAs, and the optimal nucleotide composition for a highly active gRNA could be different between SpCas9 and SaCas9. It is therefore difficult to directly compare the efficiency between SpCas9- and SaCas9-mediated gene editing with this limited number of gRNAs tested. However, it is interesting to note that the target sites for SaCas9 gRNAg and SpCas9 gRNAf have only a 1-bp shift (Fig. 1B and 2B), and the gene editing efficiencies for these two gRNAs were similar in these three *Leishmania* species. Together, these observations demonstrate that pLdSaCN can be an additional useful genome editing tool for *Leishmania* since it expands the potential PAM sequences, though, like SpCas9, the gene editing efficiency could vary significantly among different gRNAs and *Leishmania* species.

### Single-strand annealing is the predominant DSB repair mechanism for CRISPR targeting of the *MT* gene.

Based on PCR analysis of pooled CRISPR-targeted cells and a limited number of individual clones with primers closely flanking (within 2 kb) the Cas9 cleavage sites, we have previously concluded that MMEJ could mainly be responsible for DSBR in L. donovani (18, 19). To determine whether MMEJ is also present in L. major and L. mexicana, genomic DNA was extracted from the MLF-resistant cell populations for each gRNA shown in Fig. 1 and subjected to PCR analysis with primers closely flanking the Cas9 cleavage sites. Indeed, the deletion mutants specifically caused by MMEJ were detected in all these three *Leishmania* species (Fig. 1D), indicating that like in L. donovani, MMEJ is also one of the DSBR mechanisms for L. major and L. mexicana.

**FIG 3:**
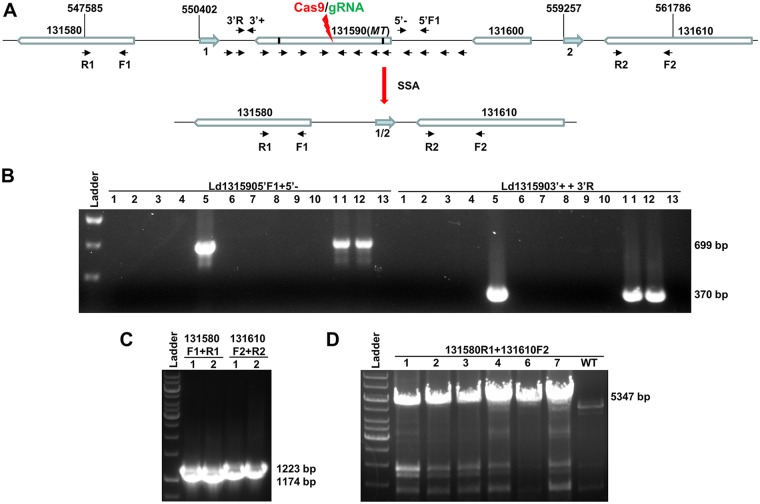
Detection of the identically sized (9-kb) large deletions caused by SSA from MLF-resistant clones after CRISPR targeting of the *MT* gene. (A) The typical genomic organization of the *Ld131590* (*MT*) gene and its adjacent genes in L. donovani chromosome 13 before and after CRISPR targeting of *MT* gene. The small vertical black lines in the *Ld131590* gene represent the microhomology sequences; the small black arrows represent the primers used to detect the deletion junction caused by CRISPR targeting and DSBR. The arrows in aqua represent the 460-bp direct repeat sequences (direct repeat sequences 1 and 2); their locations (first base) in the chromosome are indicated. Note that following CRISPR targeting of the *MT* gene and SSA-mediated DSBR, repeat 1 was merged with repeat 2 to form the single repeat (1/2). (B) PCR analysis of MLF-resistant clones with primer pair Ld1315905′F1 and 5′− and primer pair Ld1315903′+ and 3′R. Note that the expected PCR bands were detected only in clones 5, 11, and 12 among the 13 MLF-resistant clones examined. (C) PCR analysis showing that the sequences of the *Ld131590* flanking genes (*Ld131580* and *Ld131610*) were still intact in MLF-resistant clones 1 and 2. (D) PCR analysis with primer pair 131580R1 and 131610F2 revealed that a large identically sized deletion (9 kb) had occurred in all 6 MLF-resistant clones examined.

After CRISPR gene targeting, deletions ranging from 10 to 3,280 bp caused by MMEJ were previously detected in L. donovani (18) (Fig. 1D). To determine the size limit of the deletions resulting from MMEJ, the MLF-resistant promastigotes were individually cloned and subjected to PCR analysis. Interestingly, no PCR product could be detected for most of these clones using a series of primer pairs progressively farther away from the Cas9 cleavage sites (Fig. 3A and B). As an example, no PCR product could be obtained in 10 of 13 L. donovani MLF-resistant clones with primer pair Ld1315905′F1 and 5′− and primer pair Ld1315903′+R and 3′ (Fig. 3A and B). The L. donovani *131580* (*Ld131580*) and *Ld131610* genes were, however, intact in the genomes for MLF-resistant clones 1 and 2 (Fig. 3A and C). PCR primers Ld131580 R1 and Ld131610 F2 were then used to determine the location of the deletion junction in the MLF-resistant clones. Notably, a band at 5,500 bp was observed in all 6 individual MLF-resistant clones examined but not in the wild-type (WT) promastigotes, revealing that a 9-kb sequence between these primers had been deleted (Fig. 3D).

The observations presented above led us to determine whether the *MT* gene is flanked by homologous repeat sequences that could be used by single-strand annealing (SSA) to repair the DSBs, resulting in large deletions (42–46). Indeed, bioinformatic analysis revealed that the *MT* gene is flanked by two direct repeat sequences (460 and 463 bp, respectively, with 98% identities); one is in the intergenic sequence between the *Ld131580* and *MT* genes, and other is in the intergenic sequence between the *Ld131600* and *Ld131610* genes (Fig. 3A). If these direct repeat sequences were indeed used by SSA to repair the DSBs in the *MT* gene, this would result in an 8,855-bp deletion of the chromosome, generating a 5,347-bp PCR product with primers Ld131580 R1 and Ld131610 F2, as shown in Fig. 3A and D. Sequencing analysis of these PCR products confirmed that the deletion junction contained a single 460-bp repeat sequence that resulted from the merging of these two direct repeat sequences flanking the *MT* gene (Fig. 3A).

**FIG 4:**
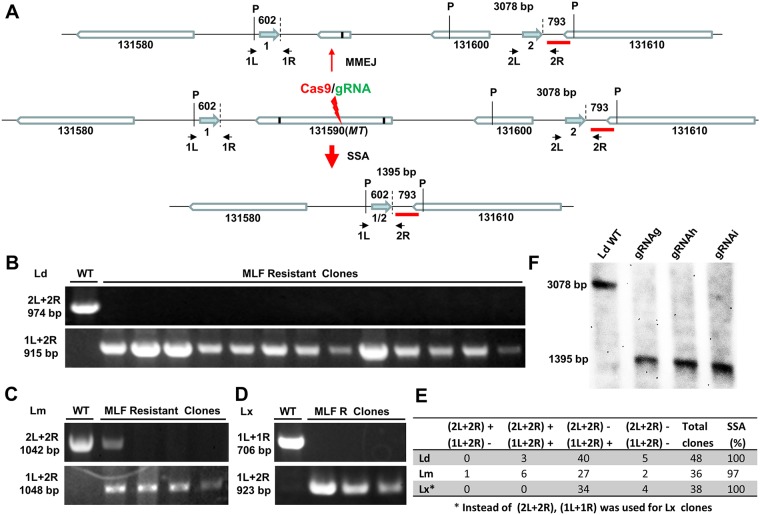
SSA was the main DSBR mechanism following CRISPR targeting of the *MT* gene in all three *Leishmania* species. (A) Genomic organization of the *Ld131590* (*MT*) gene and its adjacent genes in L. donovani chromosome 13 in the WT promastigotes (middle) or after DSBR with the MMEJ pathway (top) or the SSA pathway (bottom) following CRISPR targeting of *MT* gene. The arrows in aqua represent the 460-bp direct repeat sequences (direct repeat sequences 1 and 2); the small vertical black lines in the *Ld131590* gene represent the microhomology sequences; the primers flanking direct repeats 1 and 2, which were used to detect the SSA event, are indicated; P, PstI site; the thick red line represents the probe used for Southern blot analysis, the results of which are shown in panel F. Note that the major differences between MMEJ- and SSA-mediated DSBR are that MMEJ uses 5- to 25-nt short microhomology sequences which are frequently present in the sequences flanking the DSB. In contrast, longer homologous repeat sequences (30 to 500 nt) are required for SSA, which usually causes larger deletions. (B) Representative PCR analysis with primers 2L and 2R and primers 1L and 2R showing that SSA-mediated but no MMEJ-mediated DSBR occurred in 13 L. donovani MLF-resistant clones. (C) Representative PCR analysis with L. major-specific primers 2L and 2R and primers 1L and 2R showing that SSA- and MMEJ-mediated DSBR occurred in L. major MLF-resistant clones. (D) Representative PCR analysis with L. mexicana-specific primers 1L and 1R and primers 1L and 2R showing that SSA-mediated DSBR occurred in L. mexicana MLF-resistant clones. (E) A table summarizing the PCR analysis results for MLF-resistant clones from three different *Leishmania* species. Note that SSA accounted for more than 95% of the DSBR events in all three *Leishmania* species. (F) Southern blot analysis of genomic DNA extracted from the whole MLF-resistant cell population after CRISPR targeting of the *MT* gene in L. donovani with the DNA probe shown in panel A. Note that the 1,395-bp SSA-specific bands observed in gRNAg-, gRNAh-, and gRNAi-targeted cells had an intensity similar to that of the 3,078-bp band observed in the WT cells, and the 3,078-bp band was not detected in these CRISPR-targeted cells, indicating that SSA is the main DSBR pathway in these MLF-resistant cells.

To further investigate SSA-mediated DSB repair, we designed primers immediately flanking these direct repeat sequences (Fig. 4A). If a DSB in the *MT* gene were repaired by SSA with these repeat sequences, a PCR product of 915 bp with primer pair 1L and 2R would be detected, but it would not be detected from WT L. donovani cells or from cells where the DSB was repaired by MMEJ (Fig. 4). However, if a longer extension time were used in PCR cycles with primer pair 1L and 2R, a nearly 10-kb PCR product would be obtained for the WT cells (see Fig. S1 in the supplemental material) and a product of >915 bp but <10 kb would be obtained for the cells following MMEJ repair. In contrast, with primer pair 1L and 1R or primer pair 2L and 2R, a PCR product would be detected only in the cells with the presence of the WT allele or MMEJ-mediated repair. The presence of both the 915-bp band by PCR with primer pair 1L and 2R and the product of PCR with primer pair 1L and 1R or primer pair 2L and 2R in an MLF-resistant clone would indicate that the DSB in one chromosome was repaired by SSA and that the one in another chromosome was repaired by MMEJ (an example of an L. major MLF-resistant clone is shown in the second lane in Fig. 4C). To our surprise, as shown in Fig. 4B and E, the 915-bp band obtained with primer pair 1L and 2R (SSA band) was detected in 43 of 48 L. donovani MLF-resistant clones examined; 40 of these clones contained only the SSA band, and only 3 of these clones had both the SSA band and the band produced by PCR with primer pair 2L and 2R (MMEJ). However, 5 MLF-resistant clones did not contain the 915-bp bands obtained with primer pair 1L and 2R and primer pair 2L and 2R because these used repeats for SSA that were further away (see results below). Since the *MT* gene is similarly flanked by these repeat sequences in L. major and L. mexicana, we examined 36 L. major and 38 L. mexicana MLF-resistant clones. More than 97% of these clones had used SSA to repair the DSB, and only one clone used MMEJ alone for DSBR (Fig. 4C to E). Even more strikingly, Southern blot analysis (Fig. 4F) further revealed that the 1,395-bp SSA (PstI)-specific band with an intensity similar to that of the 3,078-bp WT (or MMEJ) band could be detected in all three genomic DNA samples extracted from the L. donovani MLF-resistant population (without cloning) targeted by gRNAg, gRNAh, and gRNAi, as described in Fig. 2. Together, these results demonstrate that SSA is the major repair mechanism for DSBs occurring in the *MT* locus and that MMEJ is not efficient in *Leishmania* since there are numerous microhomology sequences present in the 9-kb sequence between these two direct repeats.

10.1128/mSphere.00408-19.1 FIG S1 The 10-kb WT PCR product obtained with primers Ld131590 1L and 2R could be detected from WT L. donovani cells but not from the MLF-resistant cells. These PCRs were set up with a 2× KAPA *Taq* HotStart DNA polymerase mix with a 7-min extension per cycle for a total of 35 cycles. Lane 1, 1-kb DNA ladder; lane 2, WT L. donovani cells; lanes 3, 4, and 5, the pooled MLF-resistant cells from Polθ-hel(−) cells targeted with *MT* gRNAa, -b, and -c, respectively; lanes 6 and 7, MLF-resistant clones 9 and 11, respectively, from Polθ-hel(−) cells targeted with *MT* gRNAb. The 9,770-bp band in WT cells obtained with primers Ld131590 1L and 2R was detected only from WT L. donovani cells and not from the MLF-resistant cells; in contrast, the 915-bp SSA-specific band was detected only from these pooled MLF-resistant cells. Since the SSA events occurred in Polθ-hel(−) gRNAb clones 9 and 11 using repeats 3/4 and 1/4 but not 1/2, the 915-bp band was not detected in these two clones. See Results and Fig. S3 and S4 for more detail about these MLF-resistant Polθ-hel(−) cells and clones. Note that the nearly 8,000-bp band detected in the WT cells and in Polθ-hel(−) gRNAb clones 9 and 11 was a nonspecific band derived from an unknown genomic locus. Download FIG S1, TIF file, 1.4 MB. Copyright © 2019 Zhang and Matlashewski. 2019 Zhang and Matlashewski This content is distributed under the terms of the Creative Commons Attribution 4.0 International license.

### SSA could take place by using further up- and downstream direct repeat sequences resulting in larger deletions, which explains why CRISPR targeting of the adjacent *RagA* gene could lead to MLF resistance.

**FIG 5:**
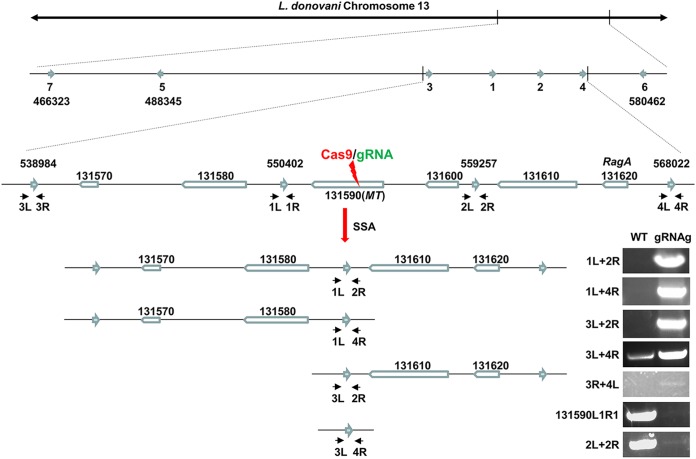
SSA could occur by using direct repeat sequences further up- and downstream, resulting in larger deletions. There were a total of 7 repeat sequences (arrows in aqua), including 5 direct repeats and 2 inverted repeats, flanking the *MT* gene within the 114-kb region in L. donovani chromosome 13. The chromosome locations of these repeats (the first base) are indicated. Genomic DNA was extracted from the MLF-resistant culture after gRNAg targeting. PCR analysis with primer pairs flanking 4 of these direct repeats adjacent to the *MT* gene showed that SSA could also occur between repeats further up- and downstream, in addition to the most common deletion between direct repeats 1 and 2. Deletions could also occur by SSA between repeats 1/4, 3/2, and 3/4, resulting in 18-, 20-, and 29-kb sequence deletions, respectively. Though with less intensity, the deletion band obtained with primers 3L and 4R between direct repeats 3 and 4 could also be detected in the WT culture, which could be attributed to the formation of the extrachromosomal circle between repeats 3 and 4, though the expected product by PCR with primers 3R and 4L was not detected in the WT cells but was detected in the MLF-resistant cells. As SSA was the main mechanism of DSBR in the *MT* gene locus, only faint bands by PCR with primer pair Ld131590 L1 and R1 and primer pair 2L and 2R were detected in this MLF-resistant cell population compared with the bands seen with WT cells. The PCR product sizes were as follows: primers 1L and 2R, 1,720 bp; primers 1L and 4R, 1,499 bp; primers 3L and 2R, 1,273 bp; primers 3L and 4R, 1,067 bp; primers 3R and 4L, 1,436 bp; primers 13150 L1 and R1, 484 bp; and primers 2L and 2R, 974 bp. The PCR bands were confirmed by sequencing.

A BLAST search revealed that there are 7 repeats flanking the *MT* gene within a 114-kb region in chromosome 13, including 5 direct and 2 inverted repeats that are 417 to 463 bp in length with 94 to 98% identities (Fig. 5). To determine whether SSA could occur between these further up- and downstream repeats, semiquantitative PCR was performed on DNA from the pooled MLF-resistant cells with primers flanking 4 of these direct repeats (Fig. 5). In addition to the main deletion event between repeats 1 and 2 described in Fig. 4, additional deletions involving further up- and downstream repeats (1/4, 3/2, and 3/4) were observed, resulting in 18-, 20-, and 29-kb deletions, respectively. This demonstrates that SSA could also arise beyond neighboring repeats, though at a lower frequency due to the proximity effect (44). This observation also supports the notion that a collision mechanism instead of the sliding mechanism was utilized by the cell to search for the complementary homologous sequences present on the 3′ ssDNA overhangs (45).

It is interesting to note that the PCR product obtained with primer pair 3L and 4R was also detected in the WT cell population, though it was less abundant in WT cells than in the CRISPR-targeted cells. This indicates that the genomic sequence (29 kb) between repeats 3 and 4 in one of the two copies of chromosome 13 was deleted in a subset of the WT cells and that this could have resulted from the formation of an extrachromosomal circle between repeats 3 and 4. Theoretically, deletions caused by the formation of extrachromosomal circles between repeats 1/2, 1/4, and 3/2 could also occur (see below), though the corresponding deletion PCR products for the 1L and 2R, 1L and 4R, and 3L and 2R primer pairs were not detected. This may suggest that it is easier for *Leishmania* to form the larger (>29-kb) circles than the smaller ones in this segment of the chromosome. Interestingly, a faint 3/4 extrachromosomal circle-specific band (obtained with primer pair 3R and 4L) was observed in the MLF-resistant cells but not in the WT cell sample, which may suggest the smaller 3/4 extrachromosomal circle formed after CRISPR targeting and DSBR could facilitate its amplification in these MLF-resistant cells (see Fig. 7 for similar data).

Based on the observations presented above, we predicted that CRISPR targeting of the *Ld131620* gene (*RagA*) could result in an *MT* gene deletion (Fig. S2). Indeed, MLF-resistant cells were detected following transfection of a CRISPR vector targeting the *RagA* gene, though at only a 10^−7^ resistance rate (1.6/10^7^ frequency). PCR analysis confirmed that the *Ld131590* (*MT*) gene was deleted, but surprisingly, the *RagA* gene remained in these MLF-resistant cells (Fig. S2C, 131620FR band). This is consistent with our recent observation that RagA is essential for *Leishmania* (unpublished data). Further PCR analysis has established the following two genomic rearrangement models which could explain how the *RagA* gene remained in these MLF-resistant cells. In the first model (Fig. S2A), a few WT *Leishmania* cells in the population already have one allele of the *MT* gene deleted due to recombination between repeats 1 and 2 under natural conditions, which was verified by detection of the product by PCR with primer pair 1L and 2R (Fig. S2C), but retain the *RagA* gene in the same chromosome. CRISPR targeting of *RagA* and subsequent SSA between repeats 1 and 4 in the other chromosome copy of these cells, as verified by detection of the product by PCR with primer pair 1L and 4R (Fig. S2C), leads to deletion of the second *RagA* gene allele together with the remaining copy of the *MT* gene, resulting in MLF resistance. In the second model (Fig. S2B), the extrachromosomal circular element containing the *Ld131610* gene and the *RagA* gene formed between repeats 2 and 4 already exists in some of these *Leishmania* cells before CRISPR targeting, and this was confirmed by detection of the product by PCR with primer pair 4L and 2R in both WT and MLF-resistant cells (Fig. S2C). Similar to the first model, CRISPR targeting of chromosomal *RagA* and SSA between repeats 1 and 4 in these cells, verified by detection of the product by PCR with primer pair 1L and 4R (Fig. S2C), leads to deletion of the *RagA* and *MT* genes in both chromosomes and MLF resistance. Together, these results reveal that the *Leishmania* genome is highly elastic and that the presence of repeat sequences flanking a target site and SSA can have dramatic effects on the end products of CRISPR targeting, which, however, can often be easily detected by the use of primers flanking these repeats. This also suggests that CRISPR targeting any of these 5 genes flanking the *MT* gene could lead to MLF resistance.

10.1128/mSphere.00408-19.2 FIG S2 Due to SSA-mediated deletion, CRISPR targeting of the upstream *Ld131620* gene (*RagA*) could lead to codeletion of the *MT* gene and MLF resistance. Two models can explain why targeting of the *Ld131620* gene led to deletion of the *Ld131590* gene, even if one copy of the *Ld131620* gene remained in the genome. In model A, one allele of the *Ld131590* gene is already deleted due to random recombination between repeats 1/2 in a few of the WT cells; SSA-mediated deletion between repeats 1 and 4 after CRISPR targeting of the *Ld131620* gene in these cells leads to deletion of the remaining *Ld131590* allele together with one copy of the *Ld131600*, *Ld131610*, and *Ld131620* genes. In model B, the extrachromosomal circle containing the *Ld131610* and *Ld131620* genes formed between repeats 2 and 4 already exists in some of the WT cells; SSA between repeats 1 and 4 during CRISPR targeting in these cells deletes both alleles of the *Ld131590* gene together with the *Ld131600*, *Ld131610*, and *Ld131620* genes present in chromosome 13. (C) The expected bands were detected for both models in PCR analysis. The PCR product sizes were as follows: primers 2L and 2R, 974 bp; primers 131620F and R, 1,118 bp; primers 1L and 4R, 1,499 bp; primers 1L and 2R, 1,720 bp; primers 4L and 2R, 1,400 bp. The PCR bands were confirmed by sequencing. Download FIG S2, TIF file, 0.7 MB. Copyright © 2019 Zhang and Matlashewski. 2019 Zhang and Matlashewski This content is distributed under the terms of the Creative Commons Attribution 4.0 International license.

As the orders of genes, including the intergenic repeat sequences, are highly syntenic in *Leishmania* species, the 7 direct and inverted repeats flanking the *MT* gene are similarly present in chromosome 13 of L. major and L. mexicana. Thus, these alternative SSA events detected in L. donovani could occur as well in L. major and L. mexicana. For example, besides the main deletion between repeats 1 and 2, the deletion between repeats 1 and 4 could also be detected in L. major MLF-resistant cells with the 1L and 4R primer pair (data not shown).

### SSA could occur between direct repeats 77 kb apart, resulting in a 15-gene codeletion.

**FIG 6:**
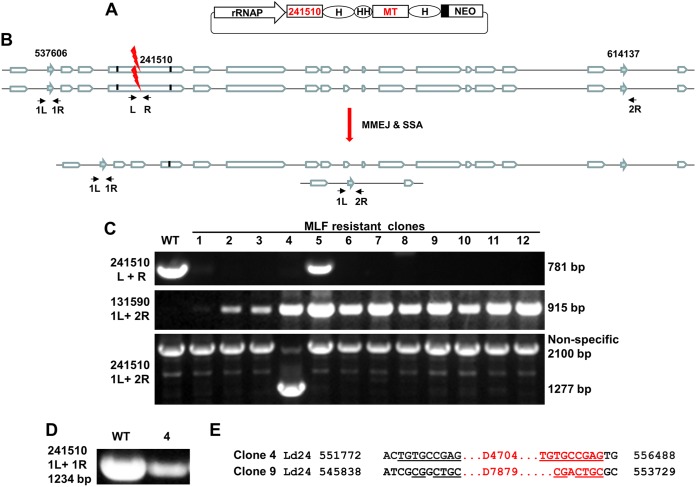
SSA could occur between direct repeats 77 kb apart, resulting in 15 *Leishmania* genes codeleted in chromosome 24. (A) The double gRNA expression plasmid pSPneogRNA241510+MT used to cotarget the *MT* gene in chromosome 13 and the *Ld241510* gene in chromosome 24. HH, hammerhead ribozyme. See the Fig. 1A legend for the definitions of the other abbreviations. (B) Genomic organization of *Ld241510* gene and its 16 adjacent genes in L. donovani chromosome 24 before and after CRISPR targeting of the *Ld241510* gene. This illustration is for clone 4 only (see below and Results). The arrows in aqua represent the 1,110-bp direct repeat sequences; the small vertical black lines in the *Ld24150* gene represent the microhomology sequences; the primers used to detect the *Ld241510* gene and the SSA event are indicated. (C) Representative PCR analysis of MLF-resistant clones after cotargeting of the *Ld241510* and *MT* genes with primers 241510 L and R, 131590 1L and 2R (as in Fig. 4), and 241510 1L and 2R. PCR analysis with primer pair 241510 1L and 2R revealed that a 77-kb sequence deletion had occurred in one of these clones (clone 4) in which the *Ld241510* and *MT* genes were cotargeted due to SSA between repeats 1 and 2 in chromosome 24. The 1,277-bp band was confirmed by sequencing. (D) PCR analysis with primers 241510 1L and 1R revealed that one repeat 1 allele remained in clone 4. (E) Sequencing analysis revealed that the remaining allele of the *Ld241510* gene in clone 4 was partially deleted (4,704 bp) by the MMEJ pathway. Note that a 7,879-bp *Ld241510* gene deletion caused by MMEJ was identified in clone 9; this was the largest MMEJ deletion detected in *Leishmania*.

A BLAST search has revealed that the *Ld241510* gene, a nonessential L. donovani species-specific gene (57), together with 14 other genes, is flanked by a pair of direct repeats (1,110 bp with 4 bp differences) 77 kb apart in chromosome 24 (Fig. 6B). To determine whether SSA-mediated DSBR could still occur between these repeats, we transfected L. donovani promastigotes with plasmids pLPhygCas9 and pSPneogRNA241510+MT (Fig. 6A), which expresses two gRNAs, one targeting the *Ld241510* gene and the other targeting the *MT* gene (19). We had previously shown that cotargeting of the *MT* and *A2* genes and selection with MLF significantly increased the *A2* gene deletion frequency (19). It was therefore interesting to determine what percentage of MLF-resistant cells had lost the *Ld241510* gene after cotargeting the *MT* gene. Indeed, out of 48 (Ld24150+MT) MLF-resistant clones examined, the *Ld241510* gene (both alleles) was deleted partially or completely (as determined by the absence of the 781-bp band by PCR with primers 241510 L and 241510 R) in 38 of these clones, and only 10 of these clones retained the *Ld24150* gene. The deletion frequency was about 80% (38/48) after cotargeting of *MT* and MLF selection (Fig. 6C). This confirmed our previous report that cotargeting of the *MT* gene could significantly improve the editing frequency of the cotargeted gene (19).

To determine whether any of these *Ld241510* gene deletions resulted from SSA between the repeats 77 kb apart, we performed PCR analysis on these *Ld241510* gene deletion clones with primers 1L and 2R flanking these repeats. Among these 38 *Ld24150* gene-deleted clones examined, one clone (clone 4) had a 77-kb sequence deletion and contained the 1,277-bp SSA-specific band (as confirmed by sequencing) (Fig. 6C). The remaining 37 clones were negative for the 1,277-bp band, indicating that the wide distance between these direct repeats did reduce the SSA frequency and that MMEJ was the main cause for *Ld241510* gene deletion in this genomic locus. Further analysis of clone 4 with primers 1L and 1R and other *Ld241510* gene primers revealed that only one allele of the *Ld241510* gene together with the other 14 genes was deleted by SSA and that deletion of the remaining *Ld241510* allele (4,704 bp) resulted from MMEJ (Fig. 6D and E). This suggests that some of the remaining 14 genes are essential for *Leishmania*. Together, these results demonstrate that SSA can occur between repeats that are 77 kb apart, leading to deletion of 15 genes in one of chromosome 24. In addition, it is interesting to note that the 7,879-bp deletion caused by MMEJ from clone 9 was the largest MMEJ-mediated deletion ever detected in *Leishmania* (Fig. 6E), further confirming that MMEJ is not efficient in *Leishmania*.

We also examined these MLF-resistant clones cotargeting *Ld24150 *and* MT* with primer pair 131590 1L and 2R and primer pair 131590 2L and 2R (Fig. 4). As expected, most of these clones were found to be positive for the 915-bp SSA-specific band in the *MT* gene locus (Fig. 6C), and only 1 of these clones was positive for the band produced by PCR with primers 131590 2L and 2R (data not shown). This further confirmed our observation, presented above, that SSA is the major DSBR pathway in the *MT* gene locus.

### DSB may prompt chromosome linear duplications.

**FIG 7:**
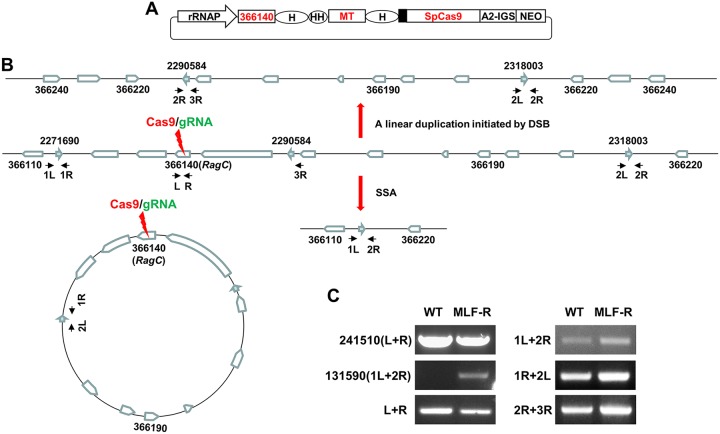
The DSB that resulted from CRISPR targeting of the *Ld366140* gene could induce a linear chromosome duplication by using nearby inverted repeat sequences. (A) The double gRNA expression CRISPR plasmid pLdCN366140+MT used to cotarget the *MT* gene in chromosome 13 and the *Ld366140* gene in chromosome 36. HH, hammerhead ribozyme. See the Fig. 1A legend for the definitions of the plasmid abbreviations. (B) The likely existing genomic rearrangements of the *Ld366140* gene locus before and after CRISPR targeting of the *Ld366140* gene, which could include the sequence deletion between direct repeats 1/2 due to the formation of the extrachromosomal circle, or by SSA induced by CRISPR targeting of the *Ld366140* gene and a linear duplication formed on inverted repeats 2 and 3. The arrows in aqua represent the 420-bp direct and inverted repeat sequences; the primers flanking these repeats, which were used to detect the various genomic rearrangements, are indicated. (C) PCR analysis confirmed the presence of these various genomic rearrangements in both WT cells and the CRISPR-cotargeted MLF-resistant cells (MLF-R). The thicker band, obtained with primers 2R and 3R, detected in MLF-resistant cells indicates that the DSB in the *Ld366140* gene could promote the linear duplication using inverted repeats 2/3. The PCR product sizes were as follows: primers 241510 L and R, 781 bp; primers 131590 1L and 2R, 915 bp; primers L and R, 371 bp; primers 1L and 2R, 1,296 bp; primers 1R and 2L, 594 bp; primers 2R and 3R, 1,184 bp. The band obtained with primers 241510 L and R was used as the PCR template control. The PCR bands were confirmed by sequencing.

We next determined whether a chromosome linear duplication can be induced by a DSB and whether SSA can occur between two direct repeat sequences alternating with one inverted repeat. CRISPR was used to cotarget the L. donovani *Ld366140* (LdBPK_366140) gene in chromosome 36 and the *MT* gene, as described above (Fig. 7A). The *Ld366140* gene is flanked by 3 repeat sequences, with 1 inverted repeat being located between 2 direct repeat sequences (Fig. 7B). These repeats are 416 to 419 bp in length and have 99% identities. However, because the RagC protein, encoded by the *Ld366140* gene, is required for healthy cell growth, though it is not essential (unpublished data), unlike cotargeting of the *Ld241510* gene, the nontargeted WT *RagC* cells could easily overgrow the *RagC* gene deletion mutants in the experiment cotargeting *MT*. As a result, the *RagC* deletion mutants could represent only a small fraction of the total MLF-resistant cell population, and this was verified by PCR analysis with *RagC*-specific primers (366140 L and R) (Fig. 7C). Nevertheless, we were interested to determine whether the 46-kb deletion could occur between direct repeats 1 and 2 by using SSA-mediated repair after targeting the *Ld366140* gene; whether a 46-kb extrachromosomal circle could be formed between repeats 1 and 2 in WT cells and, if so, whether this circle copy number could be reduced after CRISPR targeting; whether a linear duplication could occur by using inverted repeats 2 and 3; and how the copy number changed following CRISPR targeting. Surprisingly, all these three anticipated genomic rearrangements were detected by PCR analysis in both WT and CRISPR-targeted cells (Fig. 7), indicating both the extrachromosomal circular amplification resulted from genomic deletion between the direct repeats and the linear duplication obtained using the inverted repeats are commonly present for this *Ld366140* locus in the WT population. The linear duplication-specific band (obtained with primers 2R and 3R) was about 2-fold more intensive in the MLF-resistant cells than in the WT cells, indicating that CRISPR targeting of the *Ld366140* gene had increased the frequency of the linear duplication events. The deletion band seen by PCR with primers 1L and 2R was also stronger in the MLF-resistant cells than in the WT cells, strongly suggesting that SSA-mediated DSBR could still occur between direct repeats 1 and 2 after *Ld366140* gene targeting, even in the presence of an inverted repeat between them. However, instead of decreasing, the copy number of the extrachromosomal circles increased in the MLF-resistant cells (Fig. 7C). The exact reason behind this was not clear, though this finding may suggest that the smaller extrachromosomal circle resulted from CRISPR targeting and DSBR might facilitate its amplification, as seen above (Fig. 5). Lastly, since the *RagC* deletion mutants represented only a small portion of the MLF-resistant cells, the linear duplications obtained using inverted repeats 2 and 3 and deletions between direct repeats 1 and 2 could be higher in the pure *RagC* deletion mutant population than in the total MLF-resistant cells, where the *RagC* deletion mutants were mixed with WT *RagC* cells, observed here.

The *MT* gene is also flanked by two inverted repeat sequences (repeats 5 and 6; Fig. 5). Therefore, to determine whether a DSB could promote chromosome linear duplication by using these inverted repeats (Fig. S3), PCR analysis was performed with primer pair 2R and 6R or primer pair 4R and 6R on genomic DNA extracted from MLF-resistant cells after gRNAg targeting. Interestingly, a similar faint band with the expected size (972 bp) was observed with the 2R and 6R primer pair in both WT and gRNAg-targeted cells, indicating a linear duplication involving inverted repeats 2 and 6 was already present in the WT cell population. The DSB created by CRISPR gRNAg targeting did not significantly increase the frequency of this specific linear duplication (Fig. S3). In addition, no band of 747 bp was detected with primer pair 4R and 6R in these *Leishmania* cells. Additional PCR analysis with genomic DNA extracted from pLdSaCNLd131620b-transfected cells also failed to detect this linear duplication-specific band of 747 bp with primer pair 4R and 6R. This could be partly because the *Ld131620* gene is essential for *Leishmania*, which prevented the formation of this linear duplication using inverted repeats 4 and 6. Although these results show no strong evidence that CRISPR targeting prompted chromosome linear duplication in this *MT* gene locus, we did isolate an MLF-resistant clone [Polθ-hel(−) + gRNAb clone 9] which contained a linear duplication amplification by using inverted repeats 2 and 6 (see Fig. S3, results presented below, and Discussion).

10.1128/mSphere.00408-19.3 FIG S3 A linear chromosome duplication amplification observed in the *MT* gene locus. (A) The two linear duplication amplification events could occur after CRISPR targeting of the *Ld131590* (*MT*) gene. The arrows in aqua represent the 410- to 460-bp direct and inverted repeat sequences; the primers flanking these repeats, used to detect the possible linear duplications, are indicated. (B) PCR analysis revealed that a linear duplication using inverted repeats 2 and 6 but not a linear duplication using inverted repeats 4 and 6 could be present in very few of the WT cells and the MLF-resistant cells after CRISPR targeting of the *Ld131590* (*MT*) gene. A faint 972-bp band obtained with primers 6R and 2R appeared to be present in the left gel, but the 747-bp band obtained with primers 6R and 4R did not. Analysis of gRNAb targeting Polθ-hel(−) clone 9 confirmed that a linear duplication amplification in the *MT* gene locus using inverted repeats 2 and 6 could indeed occur (middle gel). The other *MT* gene allele in clone 9 was deleted by the SSA event between repeats 3 and 4 (right gel; see Fig. 5 or Fig. S4 for an illustration). The PCR bands were confirmed by sequencing. Download FIG S3, TIF file, 0.8 MB. Copyright © 2019 Zhang and Matlashewski. 2019 Zhang and Matlashewski This content is distributed under the terms of the Creative Commons Attribution 4.0 International license.

### Due to the low DSBR efficiency, more than half of DSBs created by Cas9 were not repaired in *Leishmania*, resulting in cell death.

**FIG 8:**
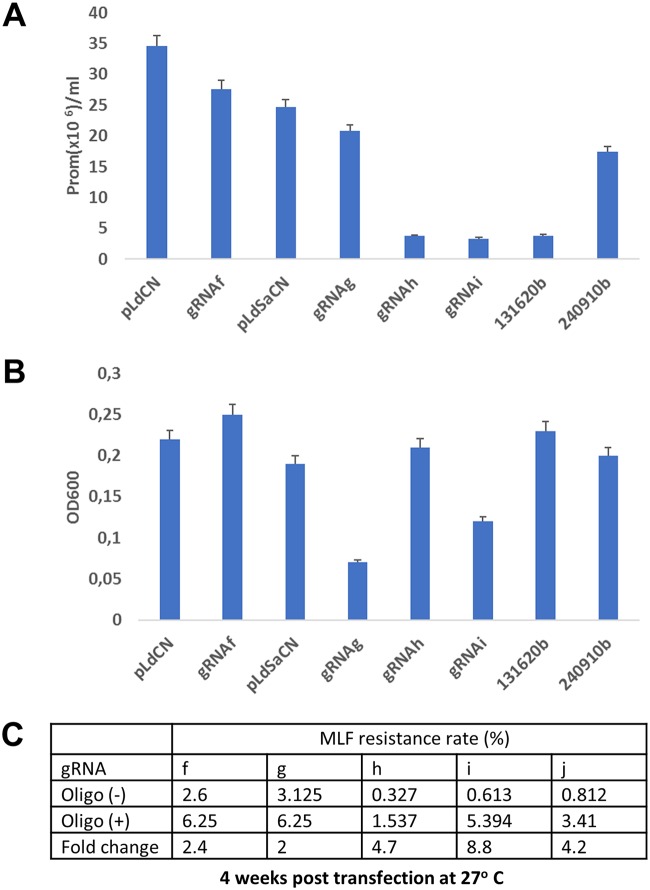
CRISPR targeting and the incompetence of DSBR had negative effects on *Leishmania* cell growth. Oligonucleotide donor transfection revealed that more than half of the DSBs were not repaired, which resulted in cell death. (A) The number of surviving *Leishmania* cells at 2 weeks posttransfection was significantly lower in cells transfected with various *Leishmania*-targeting CRISPR vectors than in cells transfected with the non-*Leishmania*-targeting control CRISPR vectors (pLdCN and pLdSaCN). The same number of *Leishmania* promastigotes (2 × 10^7^) was transfected with the same amount (5 μg) of various CRISPR vectors and selected with G418. The surviving cell number was determined at 2 weeks posttransfection. (B) The possibly selective growth of *Leishmania* cells with suboptimal CRISPR activity. The cell density for various cell lines was determined 2 days after new passages were set up with stationary-phase cells for which the results are shown in panel A. OD600, optical density at 600 nm. (C) A table showing that at least 2-fold increases of MLF resistance rates were observed in *MT* gene-targeted *Leishmania* cells after receiving the corresponding oligonucleotide DNA donors with stop codons, indicating that more than half of the DSBs created by Cas9 were not promptly repaired in *Leishmania*, which resulted in cell death. These data are representative of those from three independent transfection experiments.

**FIG 9:**
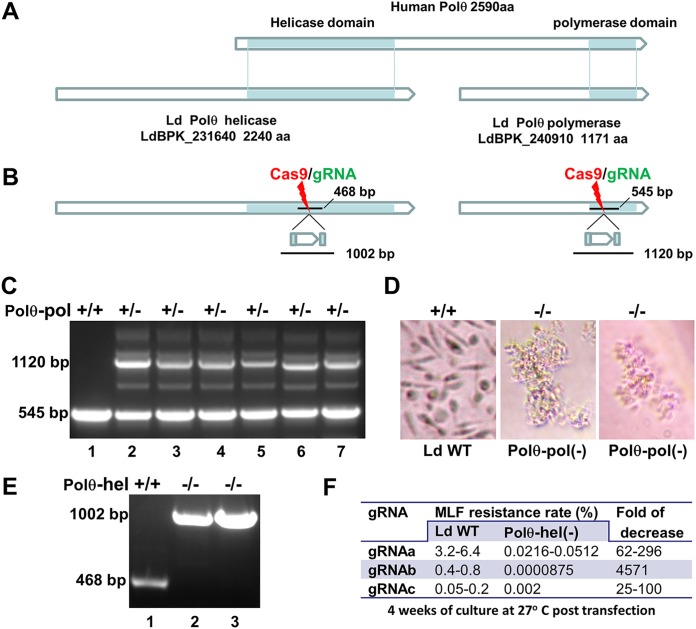
DNA Polθ polymerase is essential for *Leishmania*, and disruption of DNA Polθ helicase dramatically reduced CRISPR gene-targeting efficiency. (A) Schematic showing that, unlike human Polθ, the helicase domain and the polymerase domain of *Leishmania* are separated into two proteins. (B) CRISPR strategy used to disrupt the L. donovani Polθ helicase and Polθ polymerase genes with a bleomycin resistance gene donor. The locations of the PCR targets used to detect the disruption mutants and their sizes in WT cells and the CRISPR-targeted cells are indicated. (C) PCR analysis showing that one Polθ-pol allele was successfully disrupted with the bleomycin resistance gene donor but that the WT Polθ-pol allele band persisted in all surviving CRISPR-targeted clones examined. (D) Microscope images showing that disruption of both Polθ-pol alleles was lethal to *Leishmania*. Note that after disruption of both Polθ-pol alleles, L. donovani promastigotes continued to replicate for about 2 weeks to form clumps before final death. (E) PCR analysis showing that, unlike Polθ-pol, both Polθ-hel alleles could be successfully disrupted with the bleomycin resistance gene donor. (F) A table showing that the MLF resistance rates were dramatically decreased after CRISPR targeting of the *MT* gene in Polθ-hel null mutants compared with those in the WT L. donovani cells.

As described above, in contrast to other organisms, *Leishmania* relies on the likely most passive SSA DSBR pathway, and this leads to the overall low CRISPR gene editing rate if a donor DNA is not provided. To determine whether this results in delayed and incomplete DSBR, resulting in cell death, L. donovani promastigotes were transfected with an equal amount of different CRISPR vectors targeted to the *MT* gene and two other essential genes (the *RagA* and Polθ polymerase genes), as described in Fig. 1 and 2 and in Fig. S2 (see Fig. 9), and the control gRNA vectors with no target in the *Leishmania* genome. At 2 weeks posttransfection and after G418 selection, the number of surviving transfectants for each of these vectors was counted. As shown in Fig. 8A, compared with the control gRNA vector (pLdCN or pLdSaCN)-transfected cells, all cell lines transfected with *Leishmania* gene-targeting CRISPR vectors, regardless of the essentiality of these genes, displayed a significantly reduced cell density. Compared with the cell density for the corresponding control cell line, the cell density for gRNAf-, gRNAg-, gRNAh-, gRNAi-, Ld131620b-, and Ld240910b-expressing *Leishmania* cells was reduced to 80%, 84%, 15%, 13.5%, 15.6%, and 71%, respectively. The variation in reduced cell density could reflect the difference in activity of each gRNA and may also suggest that SaCas9 is more efficient than SpCas9 in generating DSBs. Interestingly, the pLdSaCN transfectants, which expressed SaCas9 and its control gRNA, exhibited a lower cell density (71.5%) than the SpCas9 vector pLdCN-transfected cells. This suggests that *Leishmania* cells are more tolerant of SpCas9 than of SaCas9. However, except for the gRNAg- and gRNAi-transfected cell lines, which continued to grow slowly, the other 4 cell lines started to grow like the two control cell lines when new cultures were set up after these cell lines reached stationary phase (Fig. 8B). This could be due to the selection of *Leishmania* cells with suboptimal CRISPR activity. Taken together, this demonstrates that CRISPR targeting and the inefficiency of DSBR could indeed have a negative effect on *Leishmania* cell growth. However, *Leishmania* cells may quickly develop mechanisms to escape or inhibit the CRISPR activity, for example, by selective growth of cells with lower levels of expression of Cas9 and gRNA (58, 59). This may help explain why a 100% direct gene-targeting efficiency was rarely observed even with prolonged culture (several months) after CRISPR vector transfection (Fig. 2C).

We further investigated whether some of the DSBs are not repaired, resulting in cell death in CRISPR-targeted *Leishmania* cells. The 5 *MT* gene-targeted cell lines were transfected with the corresponding oligonucleotide donor DNA containing stop codons (Table S1) and subjected to MLF selection, and the viability was determined by limiting dilution. As shown in Fig. 8C, the MLF resistance rate was increased 2- to 9-fold following oligonucleotide donor transfection compared to that in nontransfected cultures. Since oligonucleotide donor transfection could improve only DSBR and not generate more DSBs, this indicates that at least half of the DSBs were not repaired in these cultures in the absence of donor DNA, resulting in cell death. This further confirms our previous observation that oligonucleotide donor DNA transfection significantly increases the gene inactivation frequency (18, 19).

10.1128/mSphere.00408-19.6 TABLE S1 Primers and oligonucleotide donors used in this study. Download Table S1, DOCX file, 0.03 MB. Copyright © 2019 Zhang and Matlashewski. 2019 Zhang and Matlashewski This content is distributed under the terms of the Creative Commons Attribution 4.0 International license.

### DNA polymerase theta is involved in MMEJ and SSA in *Leishmania*.

DNA polymerase theta (Polθ) plays a central role in the MMEJ pathway in mammalian cells (36–41). Interestingly, unlike human polymerase (Pol), which contains an N-terminal helicase-like domain and a C-terminal polymerase domain, a BLAST search revealed two Polθ-related proteins present in *Leishmania*: one (LdBPK_231640) with 2,240 amino acids contains only the Polθ helicase domain at its C terminus (Polθ-hel), and the other (LdBPK_240910) is a 1,171-amino-acid protein and contains only the Polθ polymerase domain (Polθ-pol) (Fig. 9A). To determine whether these two Polθ-related proteins are involved in MMEJ or SSA in *Leishmania*, we attempted to disrupt both Polθ-encoding genes in L. donovani (Fig. 9B). Surprisingly, unlike in human cells, we were not able to generate the living *Leishmania* Polθ-pol null mutants. Though it was possible to disrupt one allele of the Polθ-pol gene with a bleomycin resistance gene donor (Fig. 9C), it was lethal to further disrupt the remaining Polθ-pol gene allele, as we had observed some of these CRISPR-targeted *Leishmania* cells dying within 2 weeks after cloning single allele-disrupted mutants in 96-well plates (Fig. 9D), and a 545-bp WT band persisted in all the surviving clones (Fig. 9C). We were, however, able to disrupt both *Leishmania* Polθ-hel alleles with bleomycin resistance gene donors (Fig. 9E). While these Polθ-hel-deficient cells grew normally until late log phase, they could not reach the same cell density as the WT cells in the stationary phase and were slightly shorter than the WT parasites (data not shown). To determine whether Polθ-hel deficiency would affect DSBR and CRISPR gene-targeting efficiency in *Leishmania*, we transfected the Polθ-hel-deficient cells (after removing the original Polθ-hel-targeting CRISPR vector) and WT promastigotes with CRISPR vectors expressing *MT* gene-targeting gRNAa, gRNAb, and gRNAc (18, 19) (Table S2). The MLF resistance rates were determined at 4 weeks posttransfection. As shown in Fig. 9F, compared with the WT cells, the MLF resistance rates (or *MT* diallelic mutation frequency) were dramatically reduced in all these Polθ-hel-deficient cultures targeted by gRNAa, gRNAb, or gRNAc, with the decrease being 25- to 4,500-fold.

10.1128/mSphere.00408-19.7 TABLE S2 gRNAs used in this study. Download Table S2, DOCX file, 0.01 MB. Copyright © 2019 Zhang and Matlashewski. 2019 Zhang and Matlashewski This content is distributed under the terms of the Creative Commons Attribution 4.0 International license.

**FIG 10:**
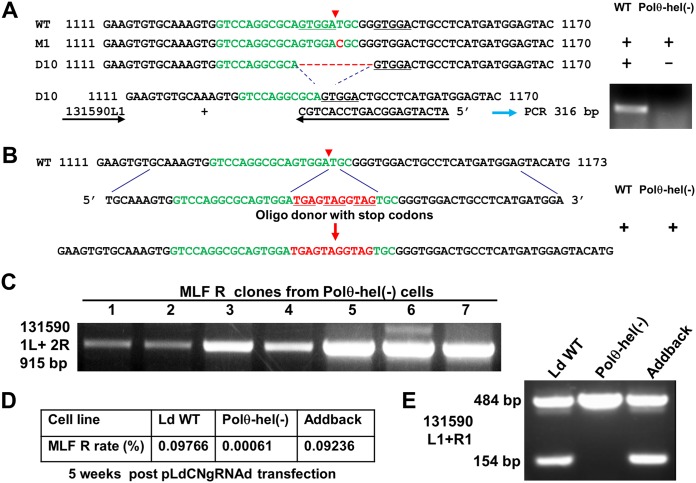
DNA Polθ-helicase is involved in both MMEJ and SSA and plays an important role in DSBR in *Leishmania*. (A) PCR and sequencing analysis showed that a single point mutation but not the typical 10-bp MMEJ deletion caused by Ld131590gRNAa targeting was detected in the Polθ-hel(−) MLF-resistant cells. (B) The oligonucleotide donor could still be used as a template to repair the DSB in the Polθ-hel(−) cells. (C) Representative PCR analysis showing that SSA remained the main DSBR pathway in the Polθ-hel(−) MLF-resistant cells, despite the markedly reduced gene editing frequency, and no MMEJ events were detected. The primer pair 131590 1L and 2R used was the same as that used in the assay whose results are shown in Fig. 4. (D) Adding back Polθ-hel to the Polθ-hel null mutant restored CRISPR gene-targeting efficiency to the level in WT cells. The Polθ-hel null mutants were first electroporated with the Polθ-hel expression vector pLPhyg-Polθ-hel. Once the cell line to which Polθ-hel was added back had been established, the cells were subsequently transfected with pLdCNgRNAd, and the MLF resistance rates were determined 5 weeks after pLdCNgRNAd transfection. (E) Adding back Polθ-hel restored the MMEJ pathway. The 154-bp band obtained with primers 131590 L1 and R1 that resulted from the 330-bp deletion caused by MMEJ (Fig. 1D) was detected in WT cells and the cell line to which Polθ-hel was added back but not in the Polθ-hel(−) cell line. Note that the 484-bp band in WT cells obtained with primers 131590 L1 and R1 was most likely derived from dead cells which contained the WT *MT* gene sequence or from some of the MLF-resistant cells which had a spontaneous mutation in the *MT* gene causing MLF resistance.

To investigate whether MMEJ could still be detected in these Polθ-hel-deficient cells, we initially examined 36 MLF-resistant clones (12 clones for each gRNA) by PCR analysis. Not unexpectedly, 27 of these clones were found to have only SSA-mediated repair (Fig. 10C). Interestingly, one of these clones contained two different SSA events involving direct repeats 1 and 4 in one chromosome 13 and a second SSA event in another chromosome 13 involving repeats 3 and 4 (Fig. S4). We also identified a clone which contained both a linear duplication event using inverted repeats 2 and 6 in one chromosome 13 and an SSA event using direct repeats 3 and 4 in another chromosome (Fig. S3). Three clones had a single point mutation at the Cas9/gRNAa cleavage site which was also detected when the WT cells were targeted, and most likely, the error was caused by HDR (Fig. 10A) (60). Interestingly, because of the extremely low CRISPR gene-targeting efficiency, many Polθ-hel-deficient parasites were screened for MLF-resistant cells, and 6 clones contained the WT sequence on the corresponding gRNA target sites, suggesting that the MLF resistance for these 6 clones was likely caused by spontaneous mutations in the *MT* gene beyond the CRISPR target sites (51, 52).

10.1128/mSphere.00408-19.4 FIG S4 Two different SSA events identified in a single MLF-resistant clone. The likely genomic organizations of the *Ld131590* (*MT*) gene and its adjacent genes in L. donovani chromosome 13 before and after CRISPR targeting of *MT* gene in the Polθ-hel(−) clone 11 are illustrated. The arrows in aqua represent the 460-bp direct repeat sequences; the primers flanking these direct repeats, used to detect these SSA events, are indicated. PCR analysis revealed that two different SSA events, one with repeats 1/4 and other with repeats 3/4, had occurred in Polθ-hel(−) clone 11. Note that the different intensities observed for these two PCR bands were likely caused by variations in amplification efficiencies with these primer pairs. Download FIG S4, TIF file, 0.5 MB. Copyright © 2019 Zhang and Matlashewski. 2019 Zhang and Matlashewski This content is distributed under the terms of the Creative Commons Attribution 4.0 International license.

Since no single MMEJ repair event was detected in any of these 36 clones, we next performed PCR analysis on genomic DNA extracted from the whole MLF-resistant cell populations without cloning. However, with this approach, apart from detection of a single point mutation at the Cas9/gRNAa cleavage site (Fig. 10A), we still could not detect any PCR bands resulted from MMEJ from the Polθ-hel-deficient MLF-resistant cell population. Since we have previously shown that a 10-bp deletion caused by microhomology sequences immediately flanking the Cas9 cleavage site in the *MT* gene could be rather easily detected in WT L. donovani promastigotes targeted by gRNAa (18), we designed a primer pair which was specific for detection of this 10-bp MMEJ deletion event. However, as shown in Fig. 10A, unlike in WT L. donovani cells, the 316-bp MMEJ-specific PCR band was absent in these pooled MLF-resistant cultures deficient in Polθ-hel. Oligonucleotide donors with only 25-nt homology arms flanking the Cas9 cleavage site have been shown to significantly improve the CRISPR gene editing efficiency in *Leishmania* (18, 19) (Fig. 8) and could be inserted by either the MMEJ, SSA, or HDR pathway. Interestingly, there was a more than 2-fold increase in MLF resistance after electroporation of the oligonucleotide donor with stop codons in these Polθ-hel-deficient cells (with the gRNAa-expressing CRISPR vector) (Fig. 10B). This suggests that *Leishmania* cells could just use HDR, SSA, or Polθ-pol alone to complete this oligonucleotide-directed DSBR. Finally, as expected, adding back of Polθ-hel to these Polθ-hel-deficient cells restored the MMEJ pathway and the CRISPR gene-targeting efficiency to the level in WT cells (Fig. 10D and E). Taken together, these results demonstrate that *Leishmania* Polθ helicase not only plays an essential role in MMEJ but also is involved in SSA, as the overall gene deletion frequencies were dramatically reduced in Polθ helicase-disrupted cells and SSA was the major DSBR mechanism in the *MT* locus. These results further confirm the importance of DSBR competence in determining CRISPR gene-targeting efficiency.

## DISCUSSION

In this study, SSA-mediated DSBR was found to account for more than 90% of the total DSBR events following CRISPR targeting in the *MT* gene locus. In contrast to our previous estimation (18, 19), only less than 10% of the DSBs were repaired through the MMEJ pathway in the same locus. It was further revealed that SSA could also use direct repeat sequences 18, 20, 29, 46, and 77 kb away to repair the DSB, though the frequency reduced with increasing distance between these direct repeats. Importantly, repeat sequences ranging from 200 to 2,500 bp, ideal substrates for SSA, are widely distributed in intergenic sequences in the *Leishmania* genome and highly conserved in *Leishmania* species (11). For example, nearly 2,000 repeats, many of which belong to short interspersed degenerate retroposons, are present in the L. major and L. infantum genomes (11). These repeats, either in a direct or in an inverted orientation, represent 5% of the *Leishmania* genome sequence and are dispersed throughout all chromosomes at a distance of between 1 and 100 kb (11). In addition, repeat sequences as small as 29 bp could be used for SSA, though homology dependence was approximately linear up to 415 bp in the yeast Saccharomyces cerevisiae (45). Besides these intergenic repeated sequences longer than 200 bp, repeated genes and short repeat sequences (26 to 200 bp) present in the *Leishmania* genome can also be substrates for SSA. We predict that SSA will likely be the main DSBR mechanism for CRISPR targeting of many of *Leishmania* genes, especially when flanking direct repeats are within 9 kb.

A substantial increase of chromosome linear duplications initiated by CRISPR-generated DSBs was observed in the *Ld366140* gene locus but not in the *Ld131590* (*MT*) gene locus (Fig. 7; see also Fig. S3 in the supplemental material). One of the explanations could be that, unlike the *Ld131590* (*MT*) gene locus, the *Ld366140* gene is first flanked by a pair of inverted repeats 19 kb apart before the 46-kb direct repeats (Fig. 7). Once a DSB was generated in the *Ld366140* gene, it would be easier (with no competition) for the inverted repeat 3 present on the 3′ single-strand overhang to fold and anneal to direct repeat 2 to initiate the chromosome linear duplication (Fig. 7). In contrast, the *Ld131590* gene is more closely flanked by 4 direct repeat sequences (within 9 to 29 kb) before the inverted repeats (Fig. 5 and Fig. S3). Therefore, these direct repeats would be predominantly used for SSA instead of a linear duplication to repair the DSBs that occurred in the *Ld131590* gene locus (Fig. 4 and 5). Besides, once a linear duplication (formation of a new chromosome) was initiated after a DSB on one side of the chromosome by folding and annealing of the 3′ single-strand overhang on the inverted repeat sequences, in order for the cell to repair the remaining broken end and survive, the other side of the chromosome would be forced to undergo the same process and form the second linear duplication (a second new chromosome). The formation of two chromosome duplications is a much longer and more complex process than a single MMEJ or SSA event, which would most likely fail and cause cell death. Therefore, we can assume that a DSB creates conditions for nearby inverted repeat sequences to form a linear duplication amplification. However, this is a long and complex process and would most likely fail and result in cell death so may not be detected. Nevertheless, since there are many inverted repeat sequences in the *Leishmania* genome (11), it may be helpful to determine whether a linear duplication has formed using inverted repeat sequences after CRISPR targeting, especially when the primers used to detect the deletion junction caused by MMEJ or SSA have failed (Fig. 7 and Fig. S3).

So far, most SSA studies were carried out in budding yeast, which greatly helped provide an understanding of the SSA mechanisms and identified important SSA cofactors, such as Rad52 and Rad59 proteins (42–46). However, the yeast utilizes HR as the main DSBR pathway (70%) and the direct repeat sequences are not widely present in the yeast genome. To make yeast a suitable SSA study model, any homologous sequences of the DSB-targeting site must first be deleted from the genome to eliminate gene conversion (HR) and direct repeat sequences must be introduced to flank the DSB site to promote SSA (42, 44, 45). Thus, in comparison to yeast, *Leishmania* would be a good natural model to study SSA as well as other DSBR pathways (see discussion below).

Our finding that *Leishmania* utilizes SSA instead of MMEJ as the major DSBR mechanism and that SSA could occur with repeats up to 77 kb apart has several implications. First, this strongly indicates that MMEJ is not efficient in *Leishmania* since there are numerous microhomology sequences (5 to 25 nt) flanking the DSBs within the 9-kb (or 77-kb) sequence between the two direct repeats (1 and 2). Since MMEJ and SSA are similar in many aspects, except that MMEJ uses short (5- to 25-nt) microhomology sequences and SSA uses longer (26- to 500-nt) homology sequences, SSA and MMEJ may share and compete for cofactors (46). It is conceivable that the low MMEJ usage in *Leishmania* is simply due to one of the inherent features of the MMEJ pathway, in that the DNA synapses formed from these short microhomology sequences are rather unstable (42). Unlike human Polθ, separation of the helicase domain and the polymerase domain from a single protein into two different *Leishmania* proteins could also influence the overall activity of *Leishmania* Polθ, a key player in MMEJ (36–41). Second, all three DSBR pathways (HDR, MMEJ, and SSA) rely on the initiation of the resection of the 5′ to 3′ ends to generate the 3′ single-strand overhangs, which can be used by HDR as RAD51 nucleofilaments to search for sequences of identical homology from the homologous chromosome for recombination, though we have previously shown that HDR was sparingly utilized for DSBR in *Leishmania* (19). The 3′ single-strand overhangs can also be directly used by MMEJ or SSA to search for and anneal the complementary homologous sequences. With such extensive 5′-to-3′-end resections (more than 4,000 nt) observed during the SSA process, it is possible that all RAD51 protein stocks in the cell for forming the ssDNA RAD51 filaments were exhausted before reaching the direct repeat complementary sequences (61), which could then anneal in a spontaneous manner or with the help of Polθ helicase (since RAD52 is not present in *Leishmania*; see discussion below) to form a stable DNA synapse to precede to subsequent steps of end joining. In other words, once the resection of the 5′ to 3′ ends initiates, the three available DSBR pathways in *Leishmania* compete for usage of the 3′ ssDNA overhangs, and the relatively efficient 5′-to-3′-end resection activity, the incompetence of MMEJ, and the extra time required for HDR to search for the homologous sequence have enabled SSA, the most passive DSBR pathway, to take the main role in *Leishmania*. Third, it was reported that 5′ exonuclease activity in end resection is about 1 to 2 nucleotides per second in yeast (43). Thus, the resection of the 5′ to 3′ ends alone could take a minimum of 1 to 9 h to complete for the 9- to 77-kb SSA deletions observed. Adding the time required to recruit and assemble those various repair cofactors, search for by a collision mechanism, and anneal the complementary single-strand repeat sequences, followed by flap trimming, gap-filling DNA synthesis, and ligation, a minimum of 2 to 10 h could be required for *Leishmania* to complete a DSBR using SSA. Together, this strongly indicates that DSBR is not efficient in *Leishmania*.

The overall low DSBR efficiency can significantly delay DSBR in *Leishmania*, and the nonpromptly repaired DSB can lead to cell cycle arrest and even cell death. Indeed, this notion was supported by our observation that *Leishmania* cells recovered much more slowly after they were transfected with *Leishmania*-targeting CRISPR vectors than after they were transfected with control vectors, and the transfection of oligonucleotide DNA donors increased the gene-targeting frequency 2- to 9-fold. This indicates that at least 50% of the DSBs created by Cas9 were not repaired, which resulted in cell death (Fig. 8). Interestingly, similar to our observation, a recent study has shown that because of the inefficient repair of DSBs, L. major was much more sensitive than Trypanosoma cruzi to radiation. L. major showed G_1_ arrest and high mortality in response to ionizing radiation. In contrast, T. cruzi was able to efficiently repair these DSBs and did not show significant cell death after exposure to the same dose of gamma irradiation (62). Furthermore, likely because of the low efficiency of DSBR pathways other than HDR, in the absence of a repair template, cell death resulting from DSBs was also observed in Trypanosoma brucei (63). The low DSBR efficiency could explain why, compared with the gene editing efficiency in other organisms, such as Trypanosoma cruzi and Toxoplasma gondii (26, 64–66), the overall CRISPR gene editing efficiency was low in *Leishmania*, albeit SaCas9 appeared to be able to generate DSBs efficiently in *Leishmania* (Fig. 8). This overall incompetence of DSBR pathways may also explain why, unlike in human cells (39), disruption of DNA Polθ polymerase was lethal to *Leishmania*.

The DSBR inefficiency could be caused by a lack or modification of some of the important DSBR cofactors in *Leishmania*. For example, due to the lack of the two important cofactors required for NHEJ, ligase IV and XRCC4, we did not detect any NHEJ-mediated DSBR in *Leishmania*. Interestingly, although *Leishmania* does not have the complete NHEJ pathway, *Leishmania* retains the Ku70/80 proteins, the other two important NHEJ cofactors (2, 47, 48). While, like in T. brucei, Ku proteins may be required for maintaining the telomere length, it is possible that this Ku heterodimer can also bind the DNA broken ends to compete, block, and slow down the resection of the 5′ to 3′ ends and so delay the process of other DSBR pathways. Indeed, it has been shown that T. brucei Ku70 and Ku80 null mutants were consistently more resistant than WT T. brucei cells to treatment with DSB-inducing reagents (67). Since NHEJ is the most efficient DSBR pathway in mammalian cells, to improve DSBR efficiency in *Leishmania* and overcome the possible hindering effect of Ku70/80 on other DSBR pathways, it will be interesting to determine whether it is possible to reconstitute the NHEJ pathway in *Leishmania* by transgenic expression of ligase IV and XRCC4 from humans or other organisms, as in yeast and Escherichia coli, where the NHEJ pathway is reconstituted by the transgenic expression of mycobacterial Ku and ligase proteins (68, 69). In mammalian cells and yeast, the Rad52 protein plays a central role in HR and SSA, as it interacts directly with both RPA and Rad51 to promote the assembly of the Rad51 nucleoprotein on ssDNA and assist with the homology search and complementary ssDNA annealing (45). Surprisingly, no Rad52 homologue is present in trypanosomatids, including *Leishmania* (2, 47), which may explain why gene targeting with the traditional homologous recombination approach was not efficient, and HDR was seldom used for DSBR in *Leishmania* (19). However, it is not clear why, unlike *Leishmania*, the other trypanosomatidic parasite T. brucei, which also lacks the Rad52 protein, has a very efficient HR pathway that requires only a 50-bp homologous sequence for gene targeting and mainly uses HDR to repair DSBs (70–73).

DNA polymerase θ is the key player in MMEJ in mammalian cells. The polymerase domain located at the C terminus of Polθ is required in MMEJ for annealing the 3′ overhangs and filling the gaps between broken DNA ends. The ATP-dependent helicase domain at the N terminus of Polθ may form a homodimer and facilitate the search for and the annealing of the microhomology sequences on the 3′ ssDNA overhangs (36–41). Indeed, we demonstrated that the Polθ polymerase is essential for *Leishmania* and that Polθ helicase not only is required for MMEJ but also plays an important role in SSA. Polθ helicase may function like the Rad52 protein to compete with and replace the RPA and Rad51 proteins bound on the ssDNAs and to help search for and anneal the complementary homologous sequences to form the stable DNA synapse. As mentioned above, unlike mammalian cells, the Polθ helicase and polymerase domains are present in two different proteins in *Leishmania*, which could affect the overall function of Polθ, though they may form a heterodimer, as some sequence similarities are present in several parts of these two proteins. Interestingly, like in *Leishmania*, the Polθ helicase and polymerase domains are also not contained in a single protein in T. cruzi. The *Leishmania* Polθ helicase is, however, much larger than the T. cruzi Polθ helicase (2,240 aa versus 998 aa) and contains a long extra N-terminal sequence (1,000 aa). Our preliminary data (not shown) suggest that the extra N-terminal sequence of *Leishmania* Polθ helicase may not be required for MMEJ but is involved in SSA. In addition, trypanosomatid Polθ helicases appear to lack the Rad51 binding domain found in human Polθ. Lastly, despite the overall differences in DSBR efficiencies among these three trypanosomatidic parasites, *Leishmania*, T. brucei, and T. cruzi, these parasites have a very similar repertoire of various DSBR pathway cofactors. For example, they all lack the NHEJ pathway and the Rad52 protein, and repeat sequences are also widely present in the T. brucei and T. cruzi genomes (2, 47–50). SSA has been observed in T. brucei (74) and could also play a role in DSBR in T. cruzi, which could explain the difficulties encountered in detecting the CRISPR deletion junctions in T. cruzi (65, 66).

Although no gRNA programs tested in this study could correctly predict SpCas9 gRNA activity in *Leishmania*, the actual activity rank order of the MLF resistance rates for these gRNAs (gRNAf > gRNAe > gRNAd) was consistent in these *Leishmania* species. This indicates that the gRNA sequence does play a role in determining its final gene-targeting efficiency. The different activity observed for the same gRNA construct in different species in the order L. mexicana > L. donovani > L. major could be due to variation in rRNA promoter activity, Cas9 nuclear localization, DSBR efficiency, and other factors in these species. Most current gRNA design programs were developed based on data from higher eukaryotes, which are normally cultured at 37°C (53–56). In contrast, the optimal culture temperature for *Leishmania* promastigotes is 27°C. This 10°C difference in culture temperature could significantly alter the binding kinetic between a gRNA and its genomic target sequence and Cas9 activity and so could affect its gene-targeting efficiency. This, together with the overall low efficiency of DSBR in *Leishmania*, could make it difficult for gRNA design programs to correctly predict gRNA activity in *Leishmania*. Nevertheless, the gRNA activity data presented here, together with future studies, could eventually help to develop a better gRNA design program for *Leishmania*.

Our finding that SSA is one of the main DSBR pathways in *Leishmania* has several important practical implications. First, as mentioned above, because direct repeat sequences are widely distributed in the *Leishmania* genome, for CRISPR targeting without using a donor DNA, it is important to determine whether the target site is flanked by direct repeat sequences (11). If so, it will be necessary to design primer pairs (example, primer pair 1L and 2R in Fig. 4) to determine whether SSA takes place after CRISPR targeting. It is also important to consider that a gene(s) in addition to the target gene deleted by SSA could contribute to a specific phenotype. This is particularly relevant when interpreting the CRISPR knockout library screening data, where many genes are likely cotargeted because of SSA. Gene complementation by the individual deleted gene can help to identify the responsible gene(s). Second, to avoid the unwanted adjacent gene deletions by SSA and improve the gene-targeting efficiency, oligonucleotide or selection marker donor DNA should be used to disrupt or delete the target gene more specifically. Finally, the direct repeat sequences (>400 bp) used for SSA could make it more difficult to detect the deletion mutations, including offsite mutations, by whole-genome Illumina shotgun sequencing due to its short reads.

In summary, this study has revealed that SSA is one of the major DSBR mechanisms in *Leishmania*. As repeat sequences are widely distributed in the genome, *Leishmania* could be an ideal model for studying the mechanism of SSA as well as other DBSR pathways. This study has revealed that CRISPR genome editing relies not only on the efficient induction of specific DSBs but also on their rapid repair through the cellular machinery. While the Cas9 cleavage efficiency in *Leishmania* needs to be further improved, such as through better gRNA design and the testing of a more robust Cas9 nuclease, including SaCas9 in this study, it is also important to consider enhancing the DSBR efficiency in *Leishmania*. Besides using donor DNA templates for targeted repairs, it will be interesting to determine whether it is possible to reconstitute the NHEJ pathway and to improve the overall DSBR efficacy by providing *Leishmania* the missing cofactors, such as DNA ligase IV and Rad52 proteins.

## MATERIALS AND METHODS

### *Leishmania* strains and culture medium.

L. donovani 1S/Cl2D, L. major Friedlin V9, and L. mexicana (MNYC/BZ/62/M379) were used in this study. The culture medium was M199 medium (pH 7.4) supplemented with 10% heat-inactivated fetal bovine serum, 40 mM HEPES (pH 7.4), 0.1 mM adenine, 5 mg liter^−1^ hemin, 1 mg liter^−1^ biotin, 1 mg liter^−1^ biopterin, 50 U ml^−1^ penicillin, and 50 μg ml^−1^ streptomycin. *Leishmania* promastigotes were routinely cultured at 27°C and passaged to fresh medium at a 40-fold dilution once a week.

The primers and oligonucleotide donors used in this study are listed in Table S1 in the supplemental material.

### Plasmid construction.

The pSPneoSagRNAH vector was generated as follows. (i) Primers LdrRNApxho1 and LdrRNApR were used to obtain the 201-bp PCR product of the L. donovani rRNA promoter; primers SagRNAF and SagRNAR were used with the pX601 (Addgene) plasmid as the template to obtain the 144-bp PCR product of the sequence encoding S. aureus gRNA (SagRNA). Primers HDVRiboF1 and pSPneoRHind3 were used to obtain the 195-bp PCR product containing the hepatitis delta virus (HDV) ribozyme-coding sequence. (ii) Primers LdrRNApXho1 and pSPneoRHind3, along with the 3 PCR products from the first step, were used as the templates (they contained 22- to 26-bp overlapping sequences) to get the 492-bp PCR product, which consisted of the L. donovani rRNA promoter, SagRNA, and the HDV ribozyme-coding sequences. (iii) The 492-bp PCR product from the second step was digested with XhoI and HindIII and cloned into the corresponding sites of the pSPneo plasmid (75) to generate pSPneoSagRNAH.

The pNeoSagRNAH vector was generated as follows. (i) The pLPneo2 plasmid (76) was digested with HindIII and BglII to get the P-neo fragment. (ii) The P-neo fragment from the first step was inserted into the pSPneoSagRNAH vector after removing the neomycin resistance gene (neo) fragment with HindIII and BglII to generate a new SagRNA expression vector, pNeoSagRNAH.

The pLdSaCN vector was generated as follows. (i) With the pX601 plasmid as the template, primers pxSaCas9F1, pxSaCas9R1, pxSaCas9F2, and pxSaCas9R2 were used to obtain the 3,265-bp PCR product of the SaCas9-coding sequence with the internal HindIII site destroyed by a silent point mutation. This SaCas9 sequence derived from plasmid pX601 (catalog number 61591; Addgene) was humanized by use of a 53% GC content and contains the nuclear localization signal of the simian virus 40 (SV40) large T antigen at its N terminus and the bipartite nuclear localization signal from nucleoplasmin at its C terminus (25). (ii) The SaCas9 fragment from the first step was inserted into the HindIII and BamHI sites of the pNeoSagRNAH vector described above to generate the SagRNA and SaCas9 coexpression vector pLdSaCN.

The pLPhygSaCas9 vector was generated by inserting the above-described 3,265-bp SaCas9-coding sequence into the HindIII and BamHI sites of the pLPhyg2 plasmid (76).

The L. donovani DNA Polθ helicase expression vector was constructed as follows. (i) The L. donovani DNA Polθ helicase gene was PCR amplified from L. donovani genomic DNA with primers Ld231640F and Ld231640R to get the 6,723-bp gene fragment. (ii) The PCR fragment was digested with HindIII and BglII and ligated into the HindIII and BamHI sites of pLphyg2 to generate the Polθ helicase expression vector.

### gRNA design.

The gRNAs used in this study were designed manually or with the aid of the following gRNA design programs: the Eukaryotic Pathogen CRISPR guide RNA Design Tool (EuPaGDT; http://grna.ctegd.uga.edu/), which was developed on the basis of data from mammalian cells and ranks a gRNA on the basis of its activity score, off-target sites in the genome, and microhomology sequences flanking the DSB (53); Sequence Scan for CRISPR (SSC; http://cistrome.org/SSC/) and CRISPRater (https://crispr.cos.uni-heidelberg.de/), which were developed from human and mouse data and which can predict gRNA activity with only the guide sequence information (55, 56); and CRISPRscan (http://www.crisprscan.org/), which was developed on the basis of zebrafish data (54). All the gRNA sequences used in this study are listed in Table S2.

### Cloning of the gRNA guide-coding sequence into *Leishmania* CRISPR vector pLdCN or pLdSaCN.

Single gRNA guide-coding sequences were ordered as standard oligonucleotides with 5′-TTGT and 5′-AAAC overhangs. It is important to note the optimal guide length for SpCas9 gRNA is 19 or 20 nt, but for SaCas9 it is 21 nt. After phosphorylation and annealing, the gRNA guide-coding sequences were ligated into the BbsI sites of the pLdCN or pLdSaCN CRISPR vector (19) (Fig. S5). The pLdCN*366140MT* vector, containing gRNA-coding sequences targeting *Ld366140* and the *MT* gene, was constructed as follows: primers Ld366140a and LdMTb and the pSPneogRNA241510+MT plasmid, which was used as the template, were used to get the 276-bp PCR product, which was then digested with BbsI and cloned into the corresponding BbsI sites of the pLdCN vector.

10.1128/mSphere.00408-19.5 FIG S5 *Leishmania* CRISPR vector pLdSaCN and its partial sequence. (A) Schematic of pLdSaCN and its guide sequence insertion site. rRNAP, L. donovani ribosomal RNA promoter; H, HDV ribozyme. The small black box represents the 92-bp pyrimidine track. The first nucleotide of gRNA, U, is highlighted (black), as L. donovani rRNAP initiates transcription at the T residue site. The SP6 promoter (indicated) can be used as the sequencing primer to confirm the insertion. The drawing is not to scale. (B) The partial sequence of pLdSaCN, which includes the 180-bp rRNAP sequence in black and its T transcription initiation site in bold, the 83-bp SaCas9 binding RNA-coding sequence in green, and the 68-bp HDV ribozyme-coding sequence in blue. The restriction enzymes XhoI and BbsI are highlighted in red. Note that the guide sequence insertion sites for *Leishmania* CRISPR vectors pLdCN, pSPneoSagRNAH, and pNeoSagRNAH are the same as those for the pLdSaCN vector. All vectors are ampicillin resistant. Download FIG S5, TIF file, 0.6 MB. Copyright © 2019 Zhang and Matlashewski. 2019 Zhang and Matlashewski This content is distributed under the terms of the Creative Commons Attribution 4.0 International license.

### Parasite transfection.

*Leishmania* promastigotes (2 × 10^7^; middle log phase to early stationary phase) in 100 μl Tb-BSF buffer (90 mM Na_2_HPO_4_, 5 mM KCl, 0.15 mM CaCl_2_, 50 mM HEPES, pH 7.3) and 2 to 5 μg CRISPR or other vectors were used for each transfection with the Lonza Nucleofector 2b device (program U33). The transfected *Leishmania* promastigotes were selected with 50 μg/ml G418 or 100 μg/ml hygromycin on the following day. Once the CRISPR vector-transfected cell culture was established, these cells could subsequently be transfected with the donors; 8 μl 100 μM oligonucleotide donor or 2 to 4 μg of purified PCR product was used for each transfection. For a drug resistance gene donor, drug selection was started 2 to 3 days after donor transfection to allow time for donor integration into the genome. Phleomycin (50 μg/ml) was initially added, and later, phleomycin was added to 100 μg/ml if the bleomycin resistance gene donor was used. These *Leishmania* cultures could sometimes be incubated at 33°C or 37°C for 2 to 3 days to improve gene editing efficiency (18).

### Determination of MLF resistance rate and cloning of MLF-resistant cells.

For *Leishmania* promastigotes transfected with various CRISPR vectors expressing *MT*-targeting gRNA, the MLF resistance rate was determined by limiting dilution culture containing 40 μM MLF in 96-well plates. Depending on the estimate of the MLF resistance rate, 2,000 to 8 million *Leishmania* promastigotes per well were inoculated into the first column of 96-well plates in quadruplicate or all eight wells for each transfected cell line. The cells in the first column were then serially 2-fold diluted from left to right up to the last column in the plate. The MLF resistance rates (or the diallelic *MT* gene mutation frequencies) were calculated after the plates were sealed and incubated in a 27°C incubator for 2 to 3 weeks. The surviving *Leishmania* cell clones in the farthest wells after MLF selection were expanded in 24-well plates for subsequent genomic DNA extraction and PCR analysis. To increase the cloning efficiency, *Leishmania* cells were sometimes limited diluted in a direction from the top to the bottom rows of the plates, or the cells from the whole MLF-resistant population selected in a 4- to 10-ml culture flask at 3 days after MLF selection were diluted and directly inoculated into 96-well plates at 1 MLF-resistant cell per 100 μl culture medium per well.

### Bioinformatics analysis.

The repeat sequences flanking a target gene were initially identified by alignment of the up- and downstream intergenic sequences progressively away from the target gene. The located repeat sequence was then used to perform a BLAST search against the *Leishmania* genome to identify the additional repeat sequences in the respective chromosome flanking the target gene. Since there are still some gaps and assembly errors in the L. donovani BPK282A1 genome sequence in TriTrypDB (http://tritrypdb.org/tritrypdb/), except for the gene identifiers, the complete sequences of these repeats described in this study and their chromosome locations were derived from the complete L. donovani genome sequence that we recently reported (77) and were verified by comparison with the L. infantum, L. major, and L. mexicana genome sequences (78). Primers flanking these repeats were designed using the Primer3 program (http://bioinfo.ut.ee/primer3-0.4.0/). The optimal primer length was 20 nucleotides with a 60°C melting temperature.

### Genomic DNA preparation and PCR analysis.

The parasite genomic DNAs were extracted from WT *Leishmania* promastigotes and various MLF-resistant cells with the minipreparation method (79), which includes phenol-chloroform extraction and ethanol precipitation. The purity and quantity of these genomic DNA were assessed by use of a NanoDrop spectrophotometer.

The various *Taq* DNA polymerases used in this study included 2× KAPA *Taq* HotStart DNA polymerase mix (Sigma-Aldrich), All in One 2× Green PCR master mix (ZmTech Scientific, Montreal, Canada), 2× DreamTaq Green PCR master mix and 2× Platinum SuperFi PCR master mix (Thermo Fisher Scientific), and Q5 high-fidelity DNA polymerase (New England BioLabs). The PCR program was set up according to the manufacturer’s instructions with variations in annealing temperature, extension time, and the total number of PCR cycles. For semiquantitative PCR, an equal amount of genomic DNA (350 ng) was used for each 12-μl PCR mixture, with the total number of cycles ranging from 22 to 35. The PCR products were separated in a 1 to 1.5% agarose gel. The putative MMEJ-, SSA-, or linear duplication-specific bands were extracted from the gel and sent to the Genome Quebec Sequencing Center for confirmation by sequencing.

### Southern blot analysis.

The same amount of genomic DNA extracted from WT- and pLdSaCNgRNAg-, pLdSaCNgRNAh-, and pLdSaCNgRNAi-transfected MLF-resistant cells was digested to completion with the restriction enzyme PstI and separated on a 0.8% agarose gel (5 μg per lane). After denaturation and neutralization, the DNA was transferred to a nylon membrane with 20× SSC (3 M NaCl and 0.3 M Na_3_ citrate) and a stack of paper towels overnight. The membrane was prehybridized at 42°C for 2 h in a prehybridization solution, consisting of 1% SDS, 1 M NaCl, 10% dextran sulfate, and 50 μg/ml denatured salmon sperm DNA (0.2 ml/cm^2^). The primer pair Ld131610F3 and Ld131610R3 was used to generate the 615-bp probe, which was biotin labeled using a Thermo Scientific biotin DecaLabel DNA-labeling kit. The membrane was then incubated overnight at 42°C in hybridization solution, containing 1% SDS, 1 M NaCl, 10% dextran sulfate, and 50 ng/ml the biotin-labeled probe. The membrane was washed twice with 2× SSC, 0.1% SDS for 10 min each time at room temperature and with 0.1× SSC, 0.1% SDS once for 20 min at 65°C. The biotin-labeled DNA probe hybridized to the target DNA on the membrane was detected with alkaline phosphatase-conjugated streptavidin and the substrate BCIP-T (5-bromo-4-chloro-3-indolylphosphate, *p*-toluidine salt) using a biotin chromogenic detection kit (Thermo Scientific).

### Data availability.

The pLdSaCN, pLPhygSaCas9, pSPneoSagRNAH, and pNeoSagRNAH plasmids have been deposited in Addgene with accession no. 123261, 123262, 123265, and 123266, respectively.
