# Supplementary figures and images for: Single-Strand Annealing Plays a Major Role in Double-Strand DNA Break Repair following CRISPR-Cas9 Cleavage in Leishmania
Source: mSphere. 2019 Aug 21;4(4):e00408-19. doi: 10.1128/mSphere.00408-19 (PMC6706467; doi:10.1128/mSphere.00408-19)

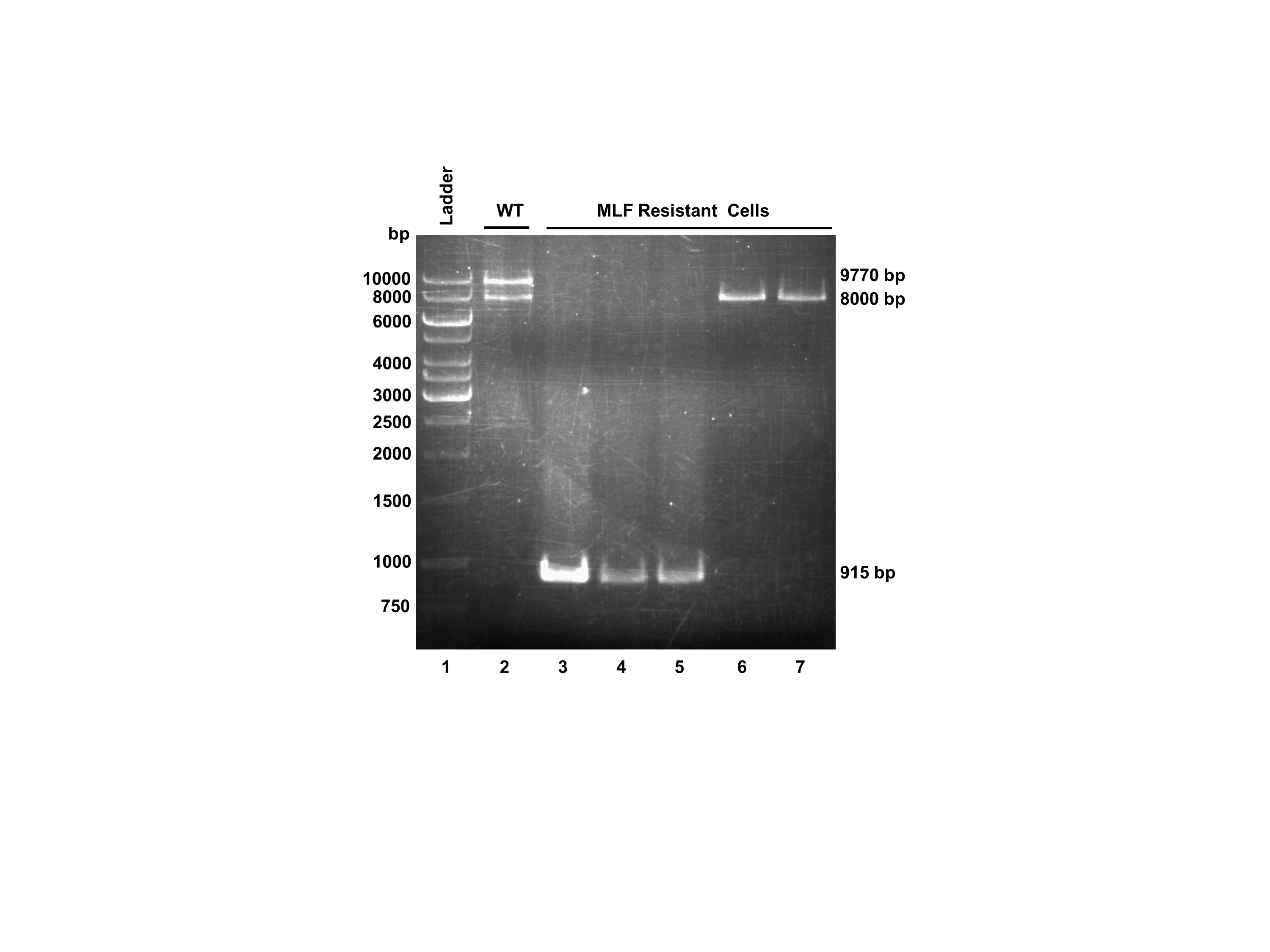

Supplement: FIG S1 [file mSphere.00408-19-sf001.tif]

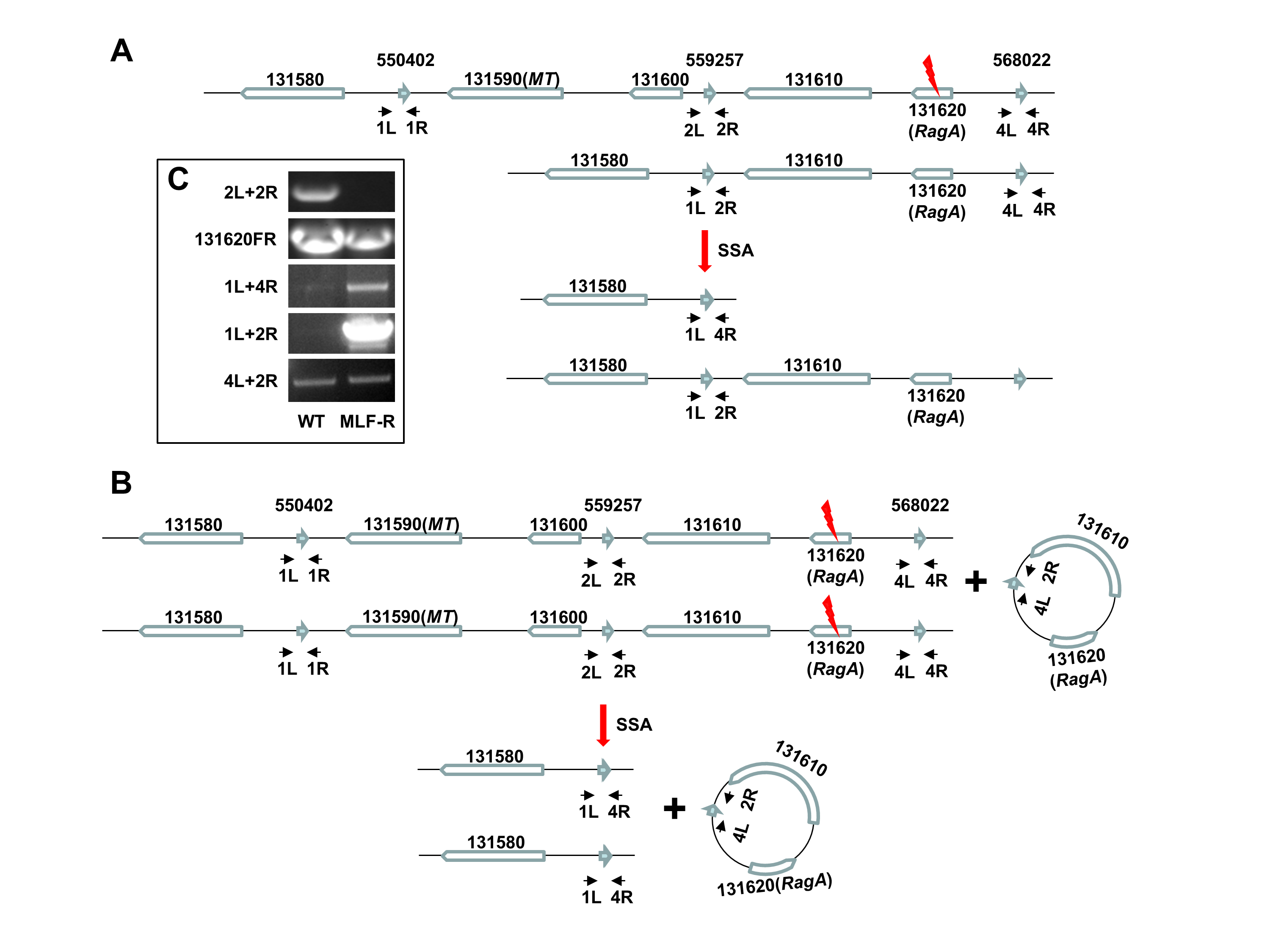

Supplement: FIG S2 [file mSphere.00408-19-sf002.tif]

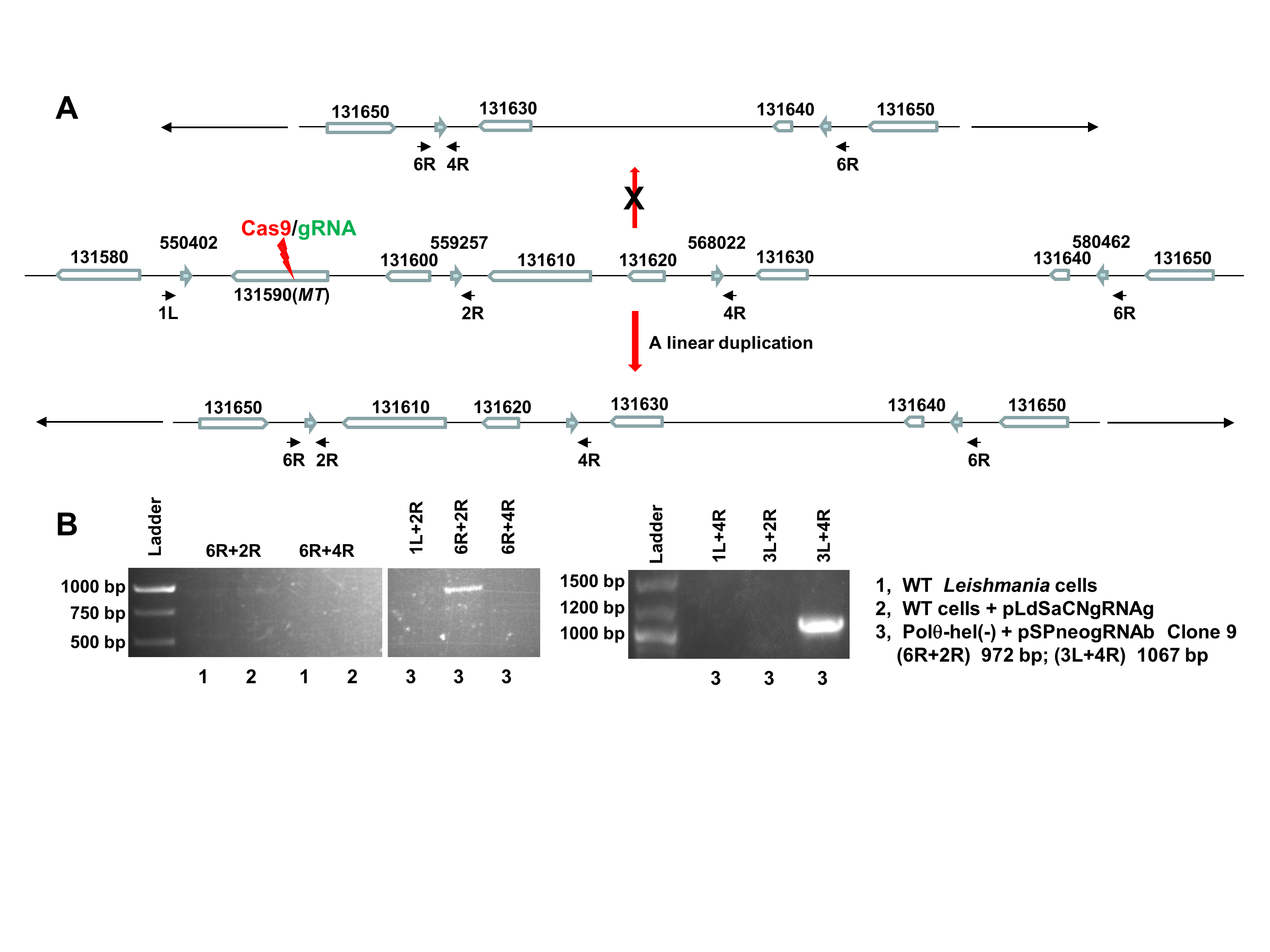

Supplement: FIG S3 [file mSphere.00408-19-sf003.tif]

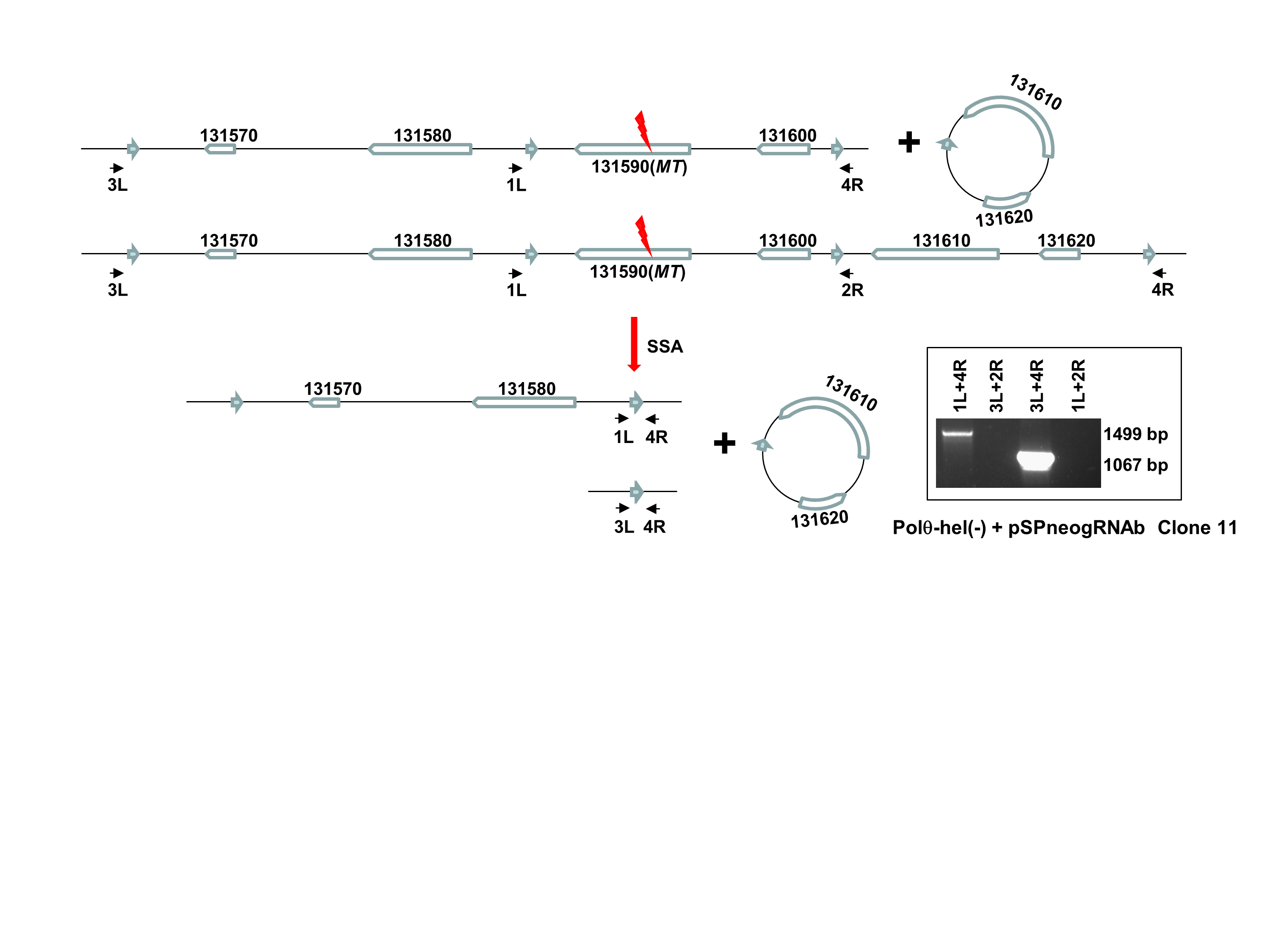

Supplement: FIG S4 [file mSphere.00408-19-sf004.tif]

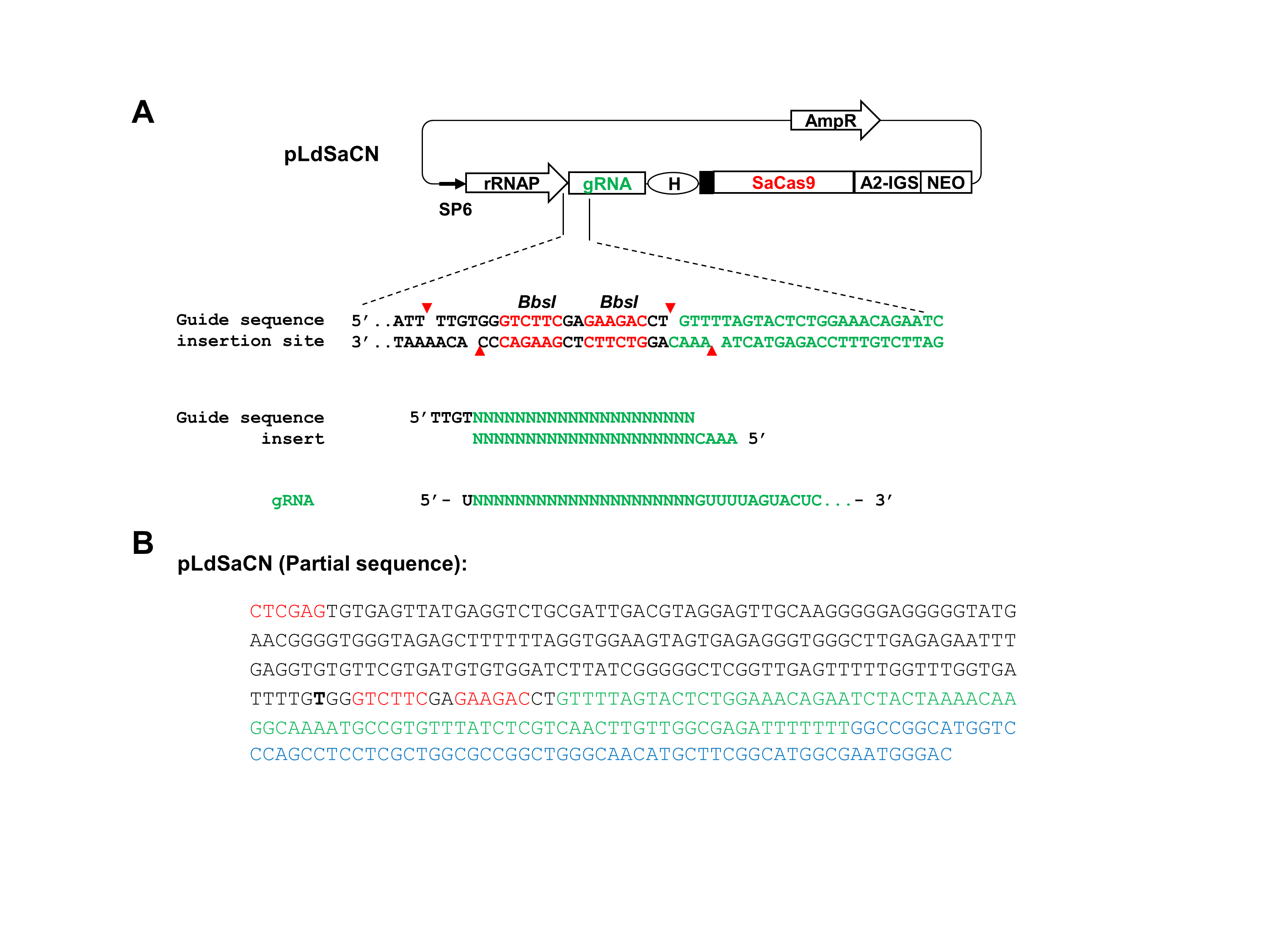

Supplement: FIG S5 [file mSphere.00408-19-sf005.tif]
