# Supplementary material for: Single-Strand Annealing Plays a Major Role in Double-Strand DNA Break Repair following CRISPR-Cas9 Cleavage in Leishmania
Source: mSphere. 2019 Aug 21;4(4):e00408-19. doi: 10.1128/mSphere.00408-19 (PMC6706467; doi:10.1128/mSphere.00408-19)
Supplement: TABLE S1 [file mSphere.00408-19-st001.docx]

**Table S1 Primers and oligonucleotide donors used in this study**

| **Fig**  **#** | **Name in Figure** | **Original**  **lab name** | **Primer sequence** |
| --- | --- | --- | --- |
| **2** |  | LdrRNApxho1 | 5’ CCCGCTCGAGTGTGAGTTATGAGGTCTGCGA |
|  |  | LdrRNApR | 5’ CTCGAAGACCCACAAAATCACCAAACCAAAAACTC |
|  |  | SagRNAF | 5’GTTTGGTGATTTTGTGGGTCTTCGAGAAGACCTGTTTTAGTACTCTGGAAACAGAATC |
|  |  | SagRNAR | 5’CAGCGAGGAGGCTGGGACCATGCCGGCCAAAAATCTCGCCAACAAGTTGA |
|  |  | HDVRiboF1 | 5’CATGGTCCCAGCCTCCTCGCTGGCGCCGGCTGGGCAACATGCTTCGG |
|  |  | pSPneoRHind3 | 5’ CTTGTTCAAGCTTGCGAATTCGAGC |
|  |  | pxSaCas9F1 | 5’ CCCAAGCTTACCATGGCCCCAAAGAAGAAGCG |
|  |  | pxSaCas9R1 | 5’ GATGCTCTGGATGAAGCTCCTCTTCACGACGGGGCTCAG |
|  |  | pxSaCas9F2 | 5’ CTGAGCCCCGTCGTGAAGAGGAGCTTCATCCAGAGCATC |
|  |  | pxSaCas9R2 | 5’ GGTAGGATCCTACTTTTTCTTTTTTGCCTGGCCGGCCTTT |
| **3** | R1 | Ld131580R1 | 5’ GAGATACCGAGCGGCTACTG |
|  | F1 | Ld131580F1 | 5’ GTACAGCGCGAGGATGTGTA |
|  | 3’R | Ld1315903’R | 5’ AAAGACGTTCCACAGCCAAC |
|  | 3’+ | Ld1315903’+ | 5’ GGTGGTGTCACTTGGGGAGGG |
|  | 5’- | Ld1315905’- | 5’ AAACGCTCAAGGCTTCCCGCGTC |
|  | 5’F1 | Ld1315905’F1 | 5’ CTTTTCCTCAGTTGCCTTGC |
|  | R2 | Ld131610R2 | 5’ CCTCAGTCGCTGAAAAGGAC |
|  | F2 | Ld131610F2 | 5’ GCCAGATATCGTGGTGGTCT |
|  | Additional primers  used | Ld131590L1 | 5’ GCTGTTCCGAAACTTGAAGC |
|  |  | Ld131590R1 | 5’ GTCGATCGGATTACCGAGAG |
|  |  | Ld131590L2 | 5’ CAGGGCTGTTGGAAATCAAT |
|  |  | Ld131590R2 | 5’ ACCATTGCTAGCTGCTCGTT |
|  |  | Ld131590L3 | 5’ CTTAACGACCCCGAGTTGAA |
|  |  | Ld131590R3 | 5’ ATGTCGATGTGGTCGATGAA |
|  |  | Ld131590L4 | 5’ TGGACGAGGATGAYGAGGTG |
|  |  | Ld131590R4 | 5’ GAATGGCGGAGTAGACGAGA |
|  |  | Ld1315905’F | 5’ CTTTTGCTTGCGTTCCTTTC |
|  |  | Ld1315905’F2 | 5’ TGTACCACCTGCAAGAGCTG |
|  |  | Ld1315905’F3 | 5’ GTATCGCCCACTTTCACGAT |
|  |  | Ld1315905’R | 5’ TGAGTGGGAGGAAGGAGATG |
|  |  | Ld1315903’F | 5’ GCTGCTTACCCGACATTGAT |
|  |  | Ld1315903’R1 | 5’ CGACTCAGTCAGCTGAGCAC |
|  |  | Ld131580F2 | 5’ CAGTAGCCGCTCGGTATCTC |
|  |  | Ld131580R2 | 5’ GGCCACTACCAAAACACGAT |
|  |  | Ld131610F1 | 5’ CAGAGGAGGCGAACTACCTG |
|  |  | Ld131610R1 | 5’ GACGACGTAGTCAGCGCATA |
| **4**  **S1** | 1L | Ld1315903’R2 | 5’ ACCTCCACCTAAACGGCTCT |
|  | 1R | Ld1315903’F1 | 5’ TGATGACCGGCGATACTTCA |
|  | 2L | Ld1316005’R | 5’ TGCACACCACTACGCGTATA |
|  | 2R | Ld1316005’F1 | 5’ TCGATCTCGTAATGCGTGAG |
|  | Lm2L | Lm1315405’R | 5’ TGCACACCGCCACAGGCGTA |
|  | Lm2R | Ld1316005’F1 | 5’ TCGATCTCGTAATGCGTGAG |
|  | Lm1L | Ld1315903’R2 | 5’ ACCTCCACCTAAACGGCTCT |
|  | Lx1L | Lx1315303’R2 | 5’ GCCTCCGCCAAAACGGCTTT |
|  | Lx1R | Ld1315903’F1 | 5’ TGATGACCGGCGATACTTCA |
|  | Lx2R | Ld1316005’F1 | 5’ TCGATCTCGTAATGCGTGAG |
|  |  | Ld131610F3 | 5’ ATCCTCTGCCGTTGGTGAA |
|  |  | Ld131610R3 | 5’ AGCAAGCAAGTCGACCTTTG |
| **5**  **S2**  **S3**  **S4** | 1L | Ld1315903’R3 | 5’ CGTACAAGAACACGCGTAGG |
|  | 1R | Ld1315903’F1 | 5’ TGATGACCGGCGATACTTCA |
|  | 2L | Ld1316005’R | 5’ TGCACACCACTACGCGTATA |
|  | 2R | Ld1316005’F1 | 5’ TCGATCTCGTAATGCGTGAG |
|  | 3L | Ld1315703’R | 5’ AAAAGACGCGAAGAGATCCA |
|  | 3R | Ld1315703’F | 5’ CATACGGAACAGGGAGTCGA |
|  | 4L | Ld1316205’R | 5’ GAGGACGATAGGGGTTAGGC |
|  | 4R | Ld1316205’F | 5’ GCCCACTTCAGCGTAGTATCA |
|  | 6R | Ld1316205’F1 | 5’ CACGCGAAGAGAAGACATGG |
|  | L1 | Ld131590L1 | 5’ GCTGTTCCGAAACTTGAAGC |
|  | R1 | Ld131590R1 | 5’ GTCGATCGGATTACCGAGAG |
|  | 131620F | Ld131620F | 5’ CCCAAGCTTCCACGCATGATTCTTCCGCGCTA |
|  | 131620R | Ld131620R | 5’ CCGGGATCCAGAACTTCGCGCATTGCACAC |
| **6** | 1L | Ld2415105’F | 5’ GCACTCACCCTTCTCCTCTT |
|  | 1R | Ld2415105’R | 5’ CAGGGAGGACGAGTGATACC |
|  | 2R | Ld2415103’R | 5’ GAGTAGGTGGTGGGTGAGTC |
|  | 241510L | Ld241510L1 | 5’ AGACGCTTTCGGCTGTACC |
|  | 241510R | Ld241510R1 | 5’ ATCAGTGCCTCCTCGTCATC |
|  | 131590  1L | Ld1315903’R2 | 5’ ACCTCCACCTAAACGGCTCT |
|  | 131590  2R | Ld1316005’F1 | 5’ TCGATCTCGTAATGCGTGAG |
|  | Additional  Primers  used | Ld241510L | 5’ GCACTTGGCATTTTTGTGG |
|  |  | Ld241510R | 5’ GAACACCTCGCGGATAATGT |
|  |  | Ld241510A | 5′ GACCCAAGCTTCCCTCTACTCGCCCTTTTCT |
|  |  | Ld241510B | 5′ GACGAGATCTCTTGACTGTCGAGTGCGTGT |
|  |  | Ld241510C | 5′ GACGGGATCCGTCGGTAATCTGCCGTTTGT |
|  |  | Ld241510D | 5′ GACGAGATCTTATGCACGTGTATGCGTTGA |
|  |  | Ld241510E | 5′ AAAGCGTGTGATGCCCTAAG |
|  |  | Ld241510G | 5′ TTATACTGCTGCTGCCGATG |
|  |  | Ld241510H | 5′ CGACCTCACATTCTTGAGCA |
|  |  | Ld2415105’F2 | 5’ GCTCACTTCGTCTTCACAGG |
|  |  | Ld2415105’F1 | 5’ CACATCATTCACCGCGACAT |
|  |  | Ld241510R3 | 5’ TAGGCTTCAACACAAACGGC |
| **7** |  | Ld366140a | 5’ATCGAAGACCTTTGTGGTCGTAGGTGCGCGACTTGGTTTTAGAGCTAGAAATAGCAAG |
|  |  | LdMTb | 5’ ATCGAAGACCCAAACGCATCCACTGCGCCTGGA |
|  | 1L | Ld3661403’R | 5’ CTAAAGGCAAACACGCAGGT |
|  | 1R | Ld3661403’F | 5’ AAGCAGGGAAACAAAGGTCG |
|  | 2L | Ld3661405’R | 5’ CAAGAGGCAGCCACGTAATC |
|  | 2R | Ld3661405’F1 | 5’ TTCTTGCTCTATTCACGCGC |
|  | 3R | Ld3661405’F | 5’ GATGTTGTGGTCCTCTCCCA |
|  | 241510L | Ld241510L1 | 5’ AGACGCTTTCGGCTGTACC |
|  | 241510R | Ld241510R1 | 5’ ATCAGTGCCTCCTCGTCATC |
|  | 131590  1L | Ld1315903’R2 | 5’ ACCTCCACCTAAACGGCTCT |
|  | 131590  2R | Ld1316005’F1 | 5’ TCGATCTCGTAATGCGTGAG |
|  | 366140L | Ld366140FC | 5’ CCGTGGACGAGCGCAACC |
|  | 366140R | Ld366140R2 | 5’ CACGTTACGGTCAATCAACG |
| **8** |  | Oligodonor f&g | 5’AACTACTTTATCATAGCCATCCTCATGAGTAGGTAGTTCCAGAACATCATGCTCTTCATCT |
|  |  | Oligodonor h | 5’CTGCTTTAGCGCAGAGGCGGACGTGAGTAGGTGACGAGGATGATGAGGTGATCGTGT |
|  |  | Oligodonor i | 5’CGCAAGGTATACAACATCCTCGCCATGAGTAGGTAGCACTGGAGTTCACGCCGGACCGCAA |
|  |  | Oligodonor j | 5’TCTTCATCTTAGCCTCCATGGCAGTGAGTAGGTAGTGGTGGAACAGCAAGTACCGGGAA |
| **9**  **10** |  | Ld240910a | 5’ATCGAAGACCTTTGTGAACCCGCAATGGACGCTGGTTTTAGAGCTAGAAATAGCAAG |
|  |  | Ld231640b | 5’ATCGAAGACCCAAACCATCGCTGCTACCCGACACCATGACGAGCTTACTC |
|  |  | Ld240910BleF1 | 5’CTTTCCCGAGAACCCGCAATGGACGCACACCAGCAACACCACAA |
|  |  | Ld240910BleR1 | 5’GAGGGGCGCAGTAGAGGTGCCGCAGGTCGGTCAGTCCTGCTCCT |
|  |  | Ld240910L | 5’GGTGTCTCGGGTGTGCTACT |
|  |  | Ld240910R | 5’GGAGGCCATACACAATCACC |
|  |  | Ld231640BleF | 5’TCCGCGTGGGTGTCGGGTAGCAGCGATCTTCATCGGATCGGGTAC |
|  |  | Ld231640BleR | 5’ACGTGCTGTGCCTCCGCCGCCGCATTCAGTCCTGCTCCTCGGCCA |
|  |  | Ld231640L | 5’GCTCTGTCACGCAGCATCT |
|  |  | Ld231640R | 5’TAGAGGTGCAGCACAGGATG |
|  |  | 131590donor | 5’TGCAAAGTGGTCCAGGCGCAGTGGATGAGTAGGTAGTGCGGGTGGACTGCCTCATGATGGA |
|  | 131590  1L | Ld1315903’R2 | 5’ ACCTCCACCTAAACGGCTCT |
|  | 131590  2R | Ld1316005’F1 | 5’ TCGATCTCGTAATGCGTGAG |
|  | 131590  L1 | Ld131590L1 | 5’ GCTGTTCCGAAACTTGAAGC |
|  | 131590  R1 | Ld131590R1 | 5’ GTCGATCGGATTACCGAGAG |
|  |  | LdMTammej | 5’ ATCATGAGGCAGTCCACTGC |
|  |  | Ld231640F | 5’ CCCAAGCTTATCGCTATGCTAAGCAGAATGCGG |
|  |  | Ld231640R | 5’ TTCAGATCTCGGCGCACACCGCGAACGCCGCCACCA |
