# Supplementary material for: Single-Strand Annealing Plays a Major Role in Double-Strand DNA Break Repair following CRISPR-Cas9 Cleavage in Leishmania
Source: mSphere. 2019 Aug 21;4(4):e00408-19. doi: 10.1128/mSphere.00408-19 (PMC6706467; doi:10.1128/mSphere.00408-19)
Supplement: TABLE S2 [file mSphere.00408-19-st002.docx]

**Table S2 A list of gRNAs used in this study**

| **Gene targeted** | **gRNA** | **Target site** |
| --- | --- | --- |
| LdBPK_131590 | gRNAa | GTCCAGGCGCAGTGGATGCGGG |
|  | gRNAb | CGCAAGAACGTAACTCTGTGGGG |
|  | gRNAc | GAAAGCTGATAAAGAAGCGGAGG |
|  | gRNAd | GTAGTTCAGCTTGCGGTCGAGG |
|  | gRNAe | ATGTTCTGGAATATGAGGATGG |
|  | gRNAf | CATGATGTTCTGGAATATGAGG |
|  | gRNAg | GAGCATGATGTTCTGGAATATGAGGAT |
|  | gRNAh | TAGCGCAGAGGCGGACGTGGACGAGGAT |
|  | gRNAi | TATACAACATCCTCGCCACACTGGAGT |
|  | gRNAj | TCTTAGCCTCCATGGCAGTGTGGTGGAA |
| LdBPK_131620 | Ld131620b | ATTGGAAGCTGCCGGAGATGCTGGAGT |
| LdBPK_241510 | Ld241510 | TGGTGTCGTTCTGCAGCCAAGG |
| LdBPK_366140 | Ld366140 | GGTCGTAGGTGCGCGACTTGAGG |
| LdBPK_231640 | Ld231640 | GTGTCGGGTAGCAGCGATGCGG |
| LdBPK_240910 | Ld240910 | GAACCCGCAATGGACGCTGCGG |
|  | Ld240910b | AGAGGTGCCGCAGCGTCCATTGCGGGT |

The PAM sequence NGG for SpCas9 and NNGRRT(A) for SaCas9 are highlighted in Green.
